# Cortical 5-HT_2A_ receptors in depression and suicide: a systematic review and meta-analysis of in vivo and post-mortem imaging studies

**DOI:** 10.1038/s41380-025-03233-4

**Published:** 2025-10-06

**Authors:** George E. Chapman, George Turner, Alexander P. Noar, Tommaso Barba, Rayyan Zafar, Robert A. McCutcheon, David Erritzoe

**Affiliations:** 1 Division of Psychiatry, Faculty of Brain Sciences, University College London, London, UK; 2 North London NHS Foundation Trust, London, UK; 3 Department of Psychiatry, University of Oxford, Oxford, UK; 4 Centre for Psychedelic Research, Imperial College London, London, UK; 5 Department of Psychosis Studies, Institute of Psychiatry, Psychology & Neuroscience, King’s College London, London, UK; 6 Oxford Health NHS Foundation Trust, Oxford, UK; 7 Central and North West London NHS Foundation Trust, London, UK

**Keywords:** Biomarkers, Diseases

## Abstract

**Introduction:**

Major depressive disorder (MDD) is a leading cause of suicide and disability. Better understanding changes to serotonin_2A_ receptors (5-HT_2A_Rs) in MDD and suicide may help to improve treatments. We systematically reviewed and meta-analysed positron emission tomography (PET), single photon emission computed tomography (SPECT) and post-mortem radioligand binding studies of cortical 5-HT_2A_Rs in MDD and suicide.

**Methods:**

Databases were searched from inception to August/September 2024. Binding data were extracted and pooled before random-effects meta-analyses of mean difference (Hedges’ *g*) and variance were undertaken. Simple linear regression was performed to investigate the relationship between receptor binding and depression severity at baseline in PET and SPECT studies. We also assessed study quality and tested for evidence of publication bias.

**Results:**

Data on 556 MDD patients or suicide victims and 526 controls from 31 studies were included. Cortical 5-HT_2A_R binding was significantly lower in living MDD patients, who had not taken antidepressants for between one week and forever, than controls in frontal, prefrontal, cingulate, anterior cingulate and, upon sensitivity analysis, temporal cortex (Hedges’ *g* = –0.40 to –0.57). In frontal and cingulate regions, binding effect size correlated with depression severity at baseline. There was study-level evidence of lower regional binding in never-medicated MDD patients than controls which, upon exploratory meta-analysis, reached significance in anterior cingulate cortex. Most PET or SPECT studies were of good or fair quality. The results of most post-mortem analyses were negative and included studies were of variable quality. There was limited evidence of publication bias.

**Conclusion:**

In vivo 5-HT_2A_R binding is reduced in MDD in frontal, cingulate and temporal cortex. This finding is based mainly on studies that used antagonist or inverse agonist radiotracers.

## Introduction

Major depressive disorder (MDD) is the world’s leading cause of suicide [1], an important cause of disability [2] and a major contributor to excess mortality seen in physical health conditions [3]. As few as one-third of MDD patients achieve remission on treatment with current first-line antidepressants [4]; however, better understanding the neurobiology of MDD could help to advance the development of treatment options. Although contested [5], the monoamine hypothesis posits that 5-hydroxytryptamine (5-HT) neurotransmission is reduced in MDD [6, 7]. Of all 5-HT neuroreceptors, the 5-HT_2A_ receptor (5-HT_2A_R) is the most highly expressed in the cortex [8] and is one of the oldest evolutionarily [9]. 5-HT_2A_Rs are mostly postsynaptic and are concentrated on the dendrites of glutamatergic pyramidal cells and some GABAergic interneurons in cortical layer V [10, 11].

5-HT_2A_R density in the living human brain can be quantified with positron emission tomography (PET) and single photon emission computed tomography (SPECT). PET and SPECT studies report binding potential (BP)—a measure of the binding of a radioligand to a given receptor. As 5-HT_2A_R ligands compete with synaptic 5-HT, 5-HT_2A_R BP reflects receptor density, radioligand affinity and endogenous 5-HT levels [12]. BP is calculated by comparing the signal from ligand bound in a brain region of interest with i) free ligand in the plasma (via an arterial input or venous output function) or ii) ligand bound in a brain region with few receptors (a reference region) [12]. An analogous technique uses a reference region approach to determine receptor density in slices or homogenates of post-mortem brain tissue [13].

Studies in humans and animals have shown that 5-HT_2A_Rs play a central role in neurotransmission, mediating cognitive and neuroendocrine functions [14, 15], and are dysregulated in psychiatric disorders [16, 17]. However, PET, SPECT and post-mortem radioligand binding studies have reached no consensus on 5-HT_2A_R alterations in MDD, reporting higher, lower or similar density to controls [18, 19]. Such mixed findings might reflect that many subjects were taking antidepressants shortly before imaging, as 5-HT_2A_R density is sensitive to alterations in synaptic 5-HT levels (and because 5-HT_1A_R agonism by antidepressants may regulate 5-HT_2A_R density directly) [20]. Additionally, some antidepressants, several neuroleptics with antidepressant properties [21] and all classical psychedelics target the 5-HT_2A_R. Psychedelics have shown promise in the treatment of depression [22, 23], where 5-HT_2A_R occupancy correlates with the intensity and mysticism of a psychedelic experience [24, 25], which may in turn predict the magnitude of antidepressant effects [26]. Meanwhile, the commonest cause of MDD patient death in many post-mortem studies—suicide—may also confound the relationship seen between depression and 5-HT_2A_R density.

We have systematically reviewed PET and SPECT studies of cortical 5-HT_2A_R binding in MDD. Two earlier reviews [27, 28] summarised differences in regional 5-HT_2A_R binding between MDD patients and healthy controls by comparing the medians and interquartile ranges of binding values from PET and SPECT studies. Our review extends this work by presenting the first meta-analysis of the PET and SPECT literature, and includes new case–control and within-subjects studies to attempt to separate illness from medication effects. We performed meta-analyses of the group variability of 5-HT_2A_R binding, which can help to interpret the results of meta-analyses of mean difference, especially if these are negative [29]. We also present the first systematic review and meta-analysis of post-mortem radioligand binding studies of cortical 5-HT_2A_R binding in MDD and suicide.

## Methods

### Registration and reporting

This review was registered with PROSPERO (CRD42019137947) in June 2019. Since registration, we decided to recruit additional reviewers to screen studies and extract data; to include studies with groups unmatched for gender; to include studies with groups unmatched for age, provided age was included as a covariate in analyses; and to systematically assess study quality. We follow the Preferred Reporting Items for Systematic reviews and Meta-Analyses (PRISMA) 2020 statement when reporting this work [30].

### Study selection

We systematically searched PubMed, EMBASE, PsycINFO and Web of Science databases from inception. We searched for all PET or SPECT studies of 5-HT_2A_R density in MDD on 8^th^ February 2024 and updated these searches on 30^th^ August 2024. We searched for all post-mortem radioligand binding studies of 5-HT_2A_R density in MDD and suicide on 6^th^ September 2024. See Supplementary Figures 1 and 2 for the full search strategy for each database.

**Fig. 1:**
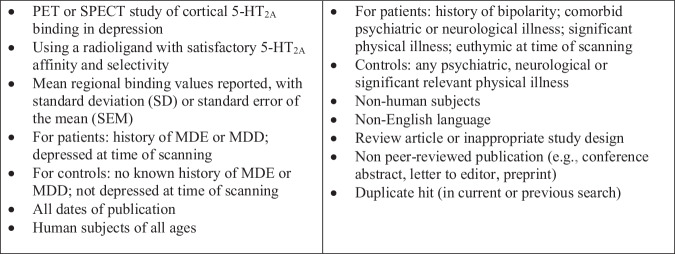
Inclusion and exclusion criteria for PET/SPECT studies of 5-HT_2A_ binding in MDD. MDD major depressive disorder, MDE major depressive episode, PET positron emission tomography, SPECT single photon emission computed tomography.

**Fig. 2:**
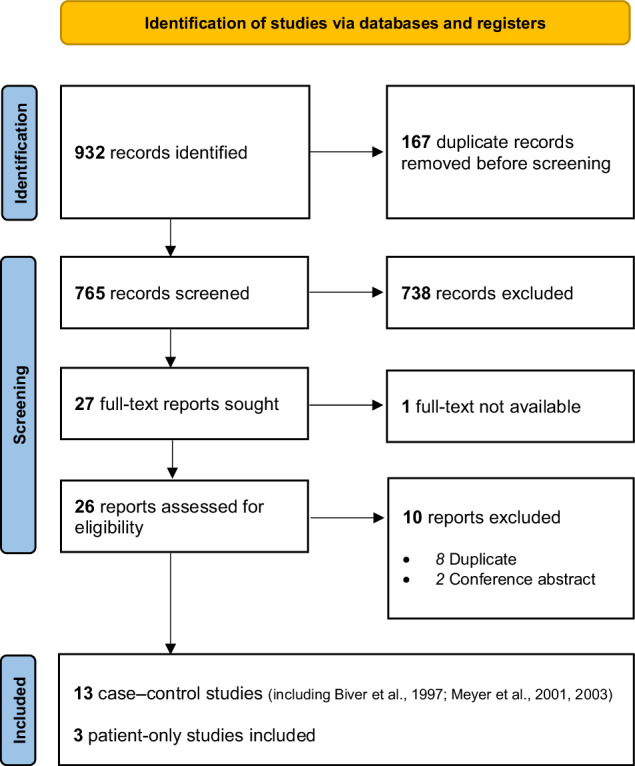
PRISMA flow-chart for finding, screening, excluding and including PET and SPECT studies of 5-HT_2A_ binding in MDD.

The titles of all identified studies, with or without abstracts, were manually screened by the lead author and one co-author (APN for PET and SPECT studies; GT for post-mortem studies), who then reviewed the full-text version of all potentially eligible studies. Any disagreements were resolved by discussion. See Figs. 1 and 2 for the inclusion and exclusion criteria and PRISMA flowchart for PET and SPECT studies. See Supplementary Figures 3–5 for the inclusion and exclusion criteria and PRISMA flowcharts for post-mortem studies. PET and SPECT searches also identified within-patient studies that looked at relationships between 5-HT_2A_R binding, antidepressant treatment and clinical outcome.

### Data collection

Mean (sub)regional binding values and standard deviations (SDs) were extracted for patient and control groups. We did not pre-specify regions for analysis; rather, all available cortical binding data were extracted. Binding data extraction was completed independently by the lead author and one co-author (APN for PET and SPECT studies; GT for post-mortem studies), who resolved any discrepancies by discussion. Additional information, such as demographic, medication and imaging details, was extracted by the lead author.

Where studies presented data for medicated and unmedicated subgroups of patients, these data were extracted and recorded separately. Where multiple studies reported binding values from the same subjects, only data from the larger study were included. Data that were only presented graphically were extracted using WebPlotDigitizer (https://automeris.io/WebPlotDigitizer/)—a semi-automated data extraction tool with high intercoder reliability and validity [31]. We contacted study authors whenever data could not be retrieved from the text or graphs. Binding data were ultimately not retrieved for three eligible PET or SPECT studies [32–34] and one eligible post-mortem study [35].

### Meta-analysis and meta-regression

(Sub)regional means and SDs were pooled. We pooled lateralised data from the left and right hemispheres. Then, if a study did not provide an overall binding value for a given region, subregional data were combined as per Supplementary Figure 6. Pooled means were calculated by taking the arithmetic mean of included means. Pooled SDs were calculated using the following formula for combining SDs of paired samples (see [36]):

$${\sigma }_{x}=\sqrt{\frac{1}{4}{{\sigma }_{a}}^{2}+\frac{1}{4}{{\sigma }_{b}}^{2}+\frac{1}{2}\rho {\sigma }_{a}{\sigma }_{b}}$$

Where ${\sigma }_{x}$ is the pooled SD; ${\sigma }_{a}$ and ${\sigma }_{b}$ are the standard deviations of the paired samples *a* and *b*; and $\rho$ is the coefficient of the correlation between samples *a* and *b*, estimated to be 0.8. When we needed to pool three or more SDs, we pooled ${\sigma }_{a}$ and ${\sigma }_{b}$ to give ${\sigma }_{x}$ then entered ${\sigma }_{x}$ into the equation with ${\sigma }_{c}$, and so on.

Meta-analyses of standardised mean difference were undertaken. We employed Hedges’ *g*, which is considered superior to other measures when sample sizes are small or uneven [37]. We used an inverse-variance random effects model due to moderate-to-high heterogeneity between some studies, as determined by the I^2^ statistic. We analysed in vivo MDD, post-mortem MDD and post-mortem suicide studies separately. A forest plot was constructed for each region for which there were case–control data available from at least three studies. We preferentially included unmedicated patient data (although most post-mortem analyses included medicated, unmedicated and mixed cohorts). If a study presented both medicated and unmedicated patient data, an additional regional forest plot was constructed containing medicated patient data. Exploratory forest plots were constructed for regions for which there were case–control data available from at least two studies in never-medicated MDD cohorts. Forest plots were also constructed to compare regional binding before and after antidepressant treatment in the same patients, wherever there were within-subjects data available from at least three studies. Finally, for post-mortem suicide studies only, we used forest plots to examine any effect of suicide method. If a study separately presented data from violent and non-violent suicides, two regional forest plots were constructed (one containing violent suicide data; one containing non-violent suicide data). If at least three studies presented both violent and non-violent suicide data for the same region, another forest plot was constructed, directly comparing violent with non-violent suicides. The Benjamini–Hochberg procedure can be used to adjust *p* values to correct for multiple comparisons. We applied this correction wherever the same binding data could have been included in two or more meta-analyses of mean difference.

We also meta-analysed group differences in the variability of 5-HT_2A_R binding, by calculating the variance ratio (VR) and coefficient of variation ratio (CVR). In accordance with published methods (see [29]), we first calculated the natural logarithm of VR and CVR—lnVR and lnCVR—which were later backtransformed to give VR and CVR. Both ratios compare within-group variance of an outcome, where VR or CVR > 1 indicates that there is greater outcome variability within the patient than control group. The larger the mean value of a biological variable, the greater the variance tends to be [38], to which VR is vulnerable but CVR is not; this is known as mean scaling [29]. We applied the Benjamini–Hochberg procedure wherever the same data could have been included in two or more meta-analyses of variance.

Negative studies are less likely to be published than positive studies, especially if they have smaller sample sizes. This publication bias was assessed using Egger’s test [39] and by visual inspection of funnel plots. The risk of bias of case–control studies was assessed using the Newcastle–Ottawa Scale ([40]; see also [41]), which involved rating the selection, comparability and exposure of cases and controls. For comparability, we considered whether studies matched or adjusted for age and gender. If ascertainment of exposure was by clinical interview, investigators need not have been blinded to case or control status for a study to score highly if other processes were robust (e.g., structured interview, independent assessment by two clinicians).

Finally, for patient groups in case–control in vivo studies, we completed meta-regressions to investigate relationships between study-level regional binding effect sizes and depression severity (mean baseline Hamilton Depression Rating Scale (HDRS) score). Data from studies using the 17- or 21-item version of the HDRS were included, as both versions should provide the same mathematical total for the same patient [42]. Simple linear regression using Pearson’s coefficient was performed for regions in which a significant difference in mean group binding was found by meta-analysis. We applied the Benjamini–Hochberg procedure wherever the same binding data could have been included in two or more meta-regressions.

All analyses were performed using the metafor package [43] within RStudio (v4.4.1, cran.r-project.org). Results were considered statistically significant if *p* < 0.05.

## Results

Our main analyses included data on 188 MDD patients and 170 controls from ten case–control PET and SPECT studies [44–53], and 349 MDD patients or suicide victims and 356 controls from 19 post-mortem studies [54–72]. Two post-mortem studies were excluded [73, 74] because their data were included in a larger study [69]. Additional within-subjects data on 19 MDD patients were included from two patient-only PET or SPECT studies [75, 76]. See Table 1 and Supplementary Tables 1 and 2 for a summary of study-level results alongside other extracted information.

**Table 1:** PET/SPECT studies: MDD versus controls.

Study	Sample	Diagnosis	Mean HDRS at baseline (SD)	Mean age (SD)	Age matched?	Gender (M:F)	Gender matched?	Medication status	Imaging modality	Radioligand; Activity at 5-HT_2A_; Dissociation constant (K_d_) at 5-HT_2A_; Test–retest data	Binding in MDD relative to controls	Any correlation between binding and medication status, depression severity or suicidality?
D’haenen et al. [47]	19 drug-free MDD	DSM-III-R for major depression	22.63 (5.37)	44.9 (14.3)	Yes	6:13	Not reported	No psychotropics for at least 1 wk; 10 patients were AD-free for at least 3 wk	SPECT	2-[123I]ketanserin; Antagonist; 1.1 nM in human cortex [107]; Test–retest data not found	↑ in bilateral PC; ( ↑ ) in superior FC, anterior PC and posterior TC; ( ↓ ) in PFC and OC; ND in CS, inferior FC and anterior TC	None found – ND in binding values between subjects off ADs for less than 1–3 wk versus more than 3 wk
	10 controls	Not reported	–	35.8 (10.8)		5:5		Not reported				–
*Biver* et al. [32]	*8 drug-free unipolar MDD*	*DSM-III-R for MDE or MDD*	*32.8 (7.3)*	*48.1 (9.7)*	*Not reported (included as covariate in analyses)*	*2:6*	*Not reported (covariate in analyses)*	*No psychotropics for at least 10 days;* *No ADs for at least 3 wk;* *Never received APs or ECT*	*PET*	*[18F]altanserin;* *Inverse agonist* *0.36 nM in human cortex* [91]; *Acceptable test–retest data* [108]	*↓ in Rt posterolateral OFC and Rt anterior insular ctx;* *(↓) in Lt posterolateral OFC and Lt anterior insular ctx*	*None found*
	*22 controls*	*No major psychiatric disorder, neurological disease or drug or alcohol misuse;* *No family history of major psychiatric disorder*	–	*38.3 (12.0)*		*12:10*		*Not reported*				*–*
Attar-Lévy et al. [45]	7 AD-free MDD	DSM-III for MDE without psychotic symptoms	54 (6)	40 (11)	Yes	3:4	Authors declined to match by pairs (prior study showed no effect of sex on setoperone binding)	6 patients were taking BZDs; No ADs for at least 2 wk (6 patients were AD-free for 1 yr)	PET	[18F]setoperone; Antagonist; 0.7 nM in rat brain [109]; Acceptable test–retest data [110]	↓ in FC; ( ↓ ) in TC and PC; ( ↑ ) in OC	None found
	7 controls	No mental or physical disease; All had normal MRI or CT head scan	–	38 (10)		4:3		“None used medication”				–
Meyer et al. [48]	14 drug-free unipolar MDD	DSM-IV for MDE secondary to MDD; No psychotic symptoms, bipolarity, other axis I disorder or alcohol or drug abuse; No suicide attempt during past 5 yr	22.5 (3.7)	32.3 (6.4)	Yes	12:2	No – more females in MDD group (*p* = 0.01) but no significant effect of age in study	No psychotropics for at least 6 mo; No non-psychotropic medication for at least 6 wk	PET	[18F]setoperone; Antagonist; 0.7 nM in rat brain [109]; Acceptable test–retest data [110]	( ↓ ) in PFC	None found – ND in binding values between subjects with and without a history of suicide attempt
	19 controls	Screened using SCI for DSM-III-R	–	31.8 (6.9)		8:11						–
Yatham et al. [49]	20 drug-free MDD	DSM-IV for major depression; No other axis I or II diagnosis; No alcohol or substance abuse within past 6 mo	27.1 (5.5)	40.1 (9.5)	Yes	9:11	Yes	No psychotropics for at least 2 wk	PET	[18F]setoperone; Antagonist; 0.7 nM in rat brain [109]; Acceptable test–retest data [110]	↓ in FC, TC, PC and OC	None found – no correlation between binding values and baseline HDRS scores
	20 controls	No history of psychiatric illness, as per SCI for DSM-III-R No first-degree relative with mood disorder or schizophrenia	–	37.2 (12.6)		8:12						–
*Meyer* et al. [33]	*19 drug-free unipolar MDD*	*DSM-IV for MDE secondary to MDD;* *No psychotic or bipolar symptoms and no other axis I diagnosis;* *No history of alcohol or drug abuse*	*21.8 (3.8)*	*30.8 (6.1)*	*Yes*	*12:7*	*Yes*	*No psychotropics for at least 3 mo;* *No ADs for at least 6 mo*	*PET*	*[18F]setoperone;* *Antagonist;* *0.7 nM in rat brain* [109]; *Acceptable test–retest data* [110];	*ND in MFG, lateral OFC, PHG, posteromedial TC and rostral ACC*	*Not reported*
	*19 controls*	*Screened using SCI for DSM-IV-R*	–	*31.8 (6.9)*		*8:11*		*No psychotropics for at least 3 mo*				*–*
**Messa et al**. [44]	**19 AD-naïve unipolar MDD**	**DSM-IV for single or recurrent MDE;** **No other axis I diagnosis;** **No alcohol or drug abuse during the past 6 mo**	**23.5 (6.3)**	**38.8 (range 21–52)**	**Yes**	**7:12**	**Yes**	**11 patients were taking BZDs;** **Never prescribed ADs, mood stabilisers or APs**	**PET**	**[18F]FESP;** **Antagonist;** **Not clear;** **Test–retest data not found**	**↓ in FC, TC, OC and ACC**	**Not reported**
	20 controls	No history of psychiatric or neurological disease; No first-degree relative with mood disorder	–	35.8 (range 22–59)		11:9		-				–
*Meyer* et al. [34]	*22 drug-free MDD*	*DSM-IV for MDE secondary to MDD;* *No bipolar disorder or other axis I disorder;* *No psychotic symptoms;* *No history of alcohol or drug abuse*	*HDRS* > *17 for inclusion*	*31* (6)	*Yes*	*Not reported*	*Not reported*	*Free of all medication for at least 5 half-lives;* *No psychotropics for at least 4 wk;* *No ADs for at least 3 mo*	*PET*	*[18F]FESP;* *Antagonist;* *Not clear;* *Test–retest data not found;*	*↑ in all regions in MDD subgroup with high (i.e., above median) dysfunctional attitude vs. controls*	*No association between binding values and baseline HDRS scores;* *Binding positively correlated with Dysfunctional Attitude Scale score*
	*29 controls*	*Screened for psychiatric illness, current suicidal ideation and history of self-harm*	–	*31* (7)								*–*
Mintun et al. [50]	46 drug-free MDD	DSM-IV for MDD; No history of neurological disorder; No other medical illness potentially affecting the CNS	23.13 (4.31)	49.6 (15.6)	Yes	16:30	Yes	No psychotropics for at least 4wk; No fluoxetine for at least 6wk	PET	[18F]altanserin Inverse agonist; 0.36 nM in human brain [91]; Acceptable test–retest data [108]	↓ in HC ( ↓ ) in pgACC, sgACC, GR, dlPFC, lTC, superior PC and OC	None found – no correlation between binding values and baseline HDRS scores in any of 8 regions tested
	29 controls	No alcohol or substance abuse; No first-degree relative with mood disorder	0.31 (0.71)	45.8 (15.3)		9:20		No psychotropics for at least 3mo				–
Sheline et al. [51]	16 drug-free MDD aged over 50 yo (subgroup from Mintun et al. [50])	DSM-IV for MDD Screened for any psychiatric illness besides depression	22.6 (3.7)	66.0 (9.5)	Yes	7:9	Yes	No psychotropics for at least 2 wk; No other “potential CNS-active drugs”	PET	[18F]altanserin; Inverse agonist; 0.36 nM in human brain [91]; Acceptable test–retest data [108]	↓ in HC (*p* = 0.05); ( ↓ ) in ACC, dlPFC, OC, GR and sgPFC; ( ↑ ) in lTC and PC	**↓ in HC in AD-naïve MDD vs. MDD with prior AD use**; No association between binding values and baseline HDRS scores
	9 controls	No history of psychiatric illness or cognitive impairment No first-degree relative with affective illness	0.4 (1.1)	64.7 (8.5)		2:7		No psychotropics for at least 4 wk or 5 half-lives; No other “potential CNS-active drugs”				–
Baeken et al. [52]	21 AD-free, medication -resistant, unipolar MDD	MINI for MDE; No alcohol or drug dependence; No suicide attempts during current episode of illness; No history of epilepsy, neurosurgery or having metal or magnetic objects in the brain	25.57 (3.92)	45.3 (11.7)	Yes	8:13	Yes	9 patients were on BZDs; No other psychotropics for at least 2 wk	SPECT	[123I]5-I-R91150; Antagonist; 0.11 nM in rat brain [111]; Test–retest data not found	↓ in Rt and Lt dlPFC; ↑ in Lt HC; ( ↑ ) in Rt HC	None found – no correlation between binding values and baseline HDRS scores
	21 controls	“Never depressed”	–	42.1 (12.6)		8:13		Free of all medication				–
**Baeken et al**. [53]	**15 AD-naïve MDD (AND)**	**DSM-IV for unipolar melancholic MDE;** **No history of bipolarity or** **alcohol or drug abuse;** **No suicide attempt during the current depressive episode**	**22.4 (6.8)**	**36.3 (9.8)**	**Yes**	**6:9**	**Yes**	**Unmedicated in current episode;** **Never prescribed ADs**	**SPECT**	**[123I]5-I-R91150;** **Antagonist;** **0.11 nM in rat brain** [111]; **Test–retest data not found**	**↓ in dorsal PFC and ACC in TRD vs. CTL;** **↓ in dorsal PFC and ACC in TRD vs. AND;** **(↓) in FC, TC, OC, ventral PFC, OFC and ACC in AND vs. CTL;** **(↑) in PC and dorsal PFC in AND vs. CTL**	**None found – post-hoc tests revealed no significant effect of current BZD or recent TCA use on binding values**
	15 treatment-resistant MDD (TRD)	DSM-IV for unipolar melancholic MDE; No history of bipolarity or alcohol or drug abuse; No suicide attempt during the current depressive episode	26.5 (3.3)	38.6 (9.5)		6:9		6 patients were on BZDs No ADs, APs or mood stabilisers for at least 2 wk				
	15 controls	“Never depressed”; No history of alcohol or drug abuse	–	37.01 (9.8)		6:9		Not reported				–
Erritzoe et al. [46]	11 AD-free MDD	MINI for MDE secondary to MDD; No other psychiatric diagnosis	21 (4)	40 (11)	No (included as covariate in analyses)	9:3	Not reported	AD-free for at least 6 mo; 6 of 12 original patients were antidepressant-naïve	PET	[11C]CIMBI-36; Agonist; 1.3 nM in rhesus monkey brain [112]; Acceptable test–retest data [113]	↑ in TC; ( ↑ ) in FC, PC and OC	Not reported
	20 controls	Not reported		32 (9)		17:3		Never previously taken psychoactive medication including SSRIs				–

↑ and ↓ denote significantly higher or lower values in MDD relative to controls, respectively. (↑) and (↓) denote non-significantly higher or lower values in MDD relative to controls, respectively.

**Studies in bold font report full data for antidepressant-naïve MDD cohort**. *Studies in italicised font were not included in meta-analyses due to unavailability of data*.

*ACC* anterior cingulate cortex; *AD(s)* antidepressant(s); *AND* antidepressant-naïve depressed; *AP(s)* antipsychotic(s); *BZD* benzodiazepines; *CIMBI-36* centre for integrated molecular brain imaging-36; *CNS* central nervous system; *CS* central sulcus; *CT* computed tomography; *ctx* cortex; *dlPFC* dorsolateral prefrontal cortex; *DSM-III(-R)* diagnostic and statistical manual version 3 (Revised); *DSM-IV* diagnostic and statistical manual version 4; *ECT* electroconvulsive therapy; *FC* frontal cortex; *FESP* fluoroethylspiperone; *GR* gyrus rectus; *HC* hippocampus; *HDRS* Hamilton depression rating scale; *lTC* lateral temporal cortex; *MDD* major depressive disorder; *MDE* major depressive episode; *MINI* Mini-international neuropsychiatric interview; *MFG* medial frontal gyrus; *mo* month(s); *MRI* magnetic resonance imaging; *ND* no statistically significant difference; *OC* occipital cortex; *OFC* orbitofrontal cortex; *PC* parietal cortex; *PET* positron emission tomography; *PFC* prefrontal cortex; *pgACC* pregenual ACC; *PHG* parahippocampal gyrus; *SCI* structured clinical interview; *sgACC* subgenual ACC; *SD* standard deviation; *sgPFC* subgenual prefrontal cortex; *SPECT* single photon emission computed tomography; *SSRI* selective serotonin reuptake inhibitor; *TC* temporal cortex; *TCA* tricyclic antidepressant; *TRD* treatment resistant depression; *wk* week(s); *yr* year(s); *yo* years old.

### PET and SPECT studies

MDD patients versus controls

#### Meta-analysis of group differences in mean binding

**Fig. 3:**
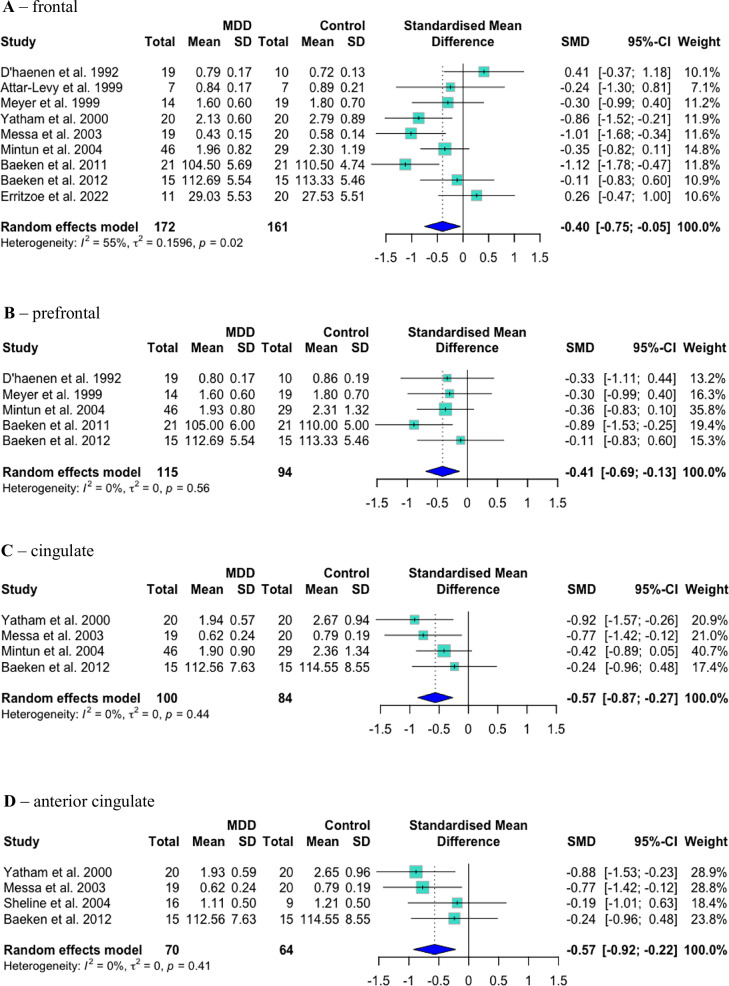
Case–control PET/SPECT studies: forest plots. Mean 5-HT_2A_ binding was significantly lower in MDD patients than controls in frontal (**A**), prefrontal (**B**), cingulate (**C**) and anterior cingulate (**D**) cortex.

**Fig. 4:**
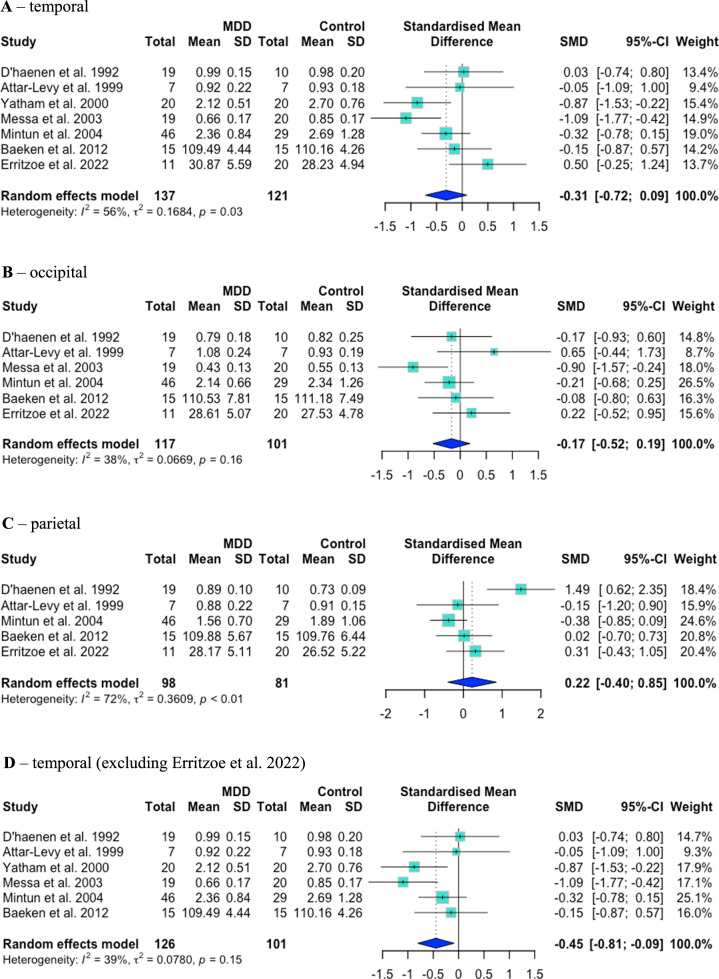
Case–control PET/SPECT studies: forest plots. Mean 5-HT_2A_ binding was not significantly different between MDD patients and controls in temporal (**A**), occipital (**B**), parietal (**C**) or temporal (not shown) cortex. When data from Erritzoe et al. 2022 were excluded from analyses, binding was significantly lower in temporal cortex (**D**).

Two studies presented separate data for medicated and unmedicated patients; however, only unmedicated patient data could be included in analyses (all medicated patients in Messa et al. [44] were euthymic; repeated measures data from Attar-Lévy et al. [45] were included in within-patient analyses). 5-HT_2A_R binding was significantly lower in unmedicated MDD patients than controls in frontal (Hedges’ *g* = –0.40, 95% CI = [–0.75, –0.05], *p*_*uncorrected*_ = 0.026, *p*_*corrected*_ = 0.039; heterogeneity I^2^ = 55%, *p* = 0.02; number of studies, *N* = 9), prefrontal (*g* = –0.41 [–0.69, –0.13], *p*_*uncorrected*_ = 0.004, *p*_*corrected*_ = 0.012; I^2^ = 0%, *p* = 0.46; *N* = 5), cingulate (*g* = –0.57 [–0.87, –0.27], *p*_*uncorrected*_ = 0.0002, *p*_*corrected*_ = 0.0006; I^2^ = 0%, *p* = 0.44; *N* = 4) and anterior cingulate (*g* = –0.57 [–0.92, –0.22], *p*_*uncorrected*_ = 0.001, *p*_*corrected*_ = 0.002; I^2^ = 0%, *p* = 0.41; *N* = 4) cortex. There were no significant differences in temporal (*g* = –0.31 [–0.72, 0.09], *p*_*uncorrected*_ = 0.13, *p*_*corrected*_ = 0.18; I^2^ = 56%, *p* = 0.03; *N* = 7), occipital (*g* = –0.17 [–0.52, 0.19], *p*_*uncorrected*_ = 0.36, *p*_*corrected*_ = 0.36; I^2^ = 38%, *p* = 0.16; *N* = 6) or parietal (*g* = 0.22 [–0.40, 0.85], *p* = 0.49; I^2^ = 72%, *p* < 0.01; *N* = 5) cortex. See Figs. 3 and 4 for seven of these forest plots.

Of the studies meta-analysed, seven used 5-HT_2A_R antagonists (2-[123I]ketanserin, [18F]setoperone, [18F]fluoroethylspiperone (FESP), [123I]5-I-R91150), two used an inverse agonist ([18F]altanserin) and one used an agonist ([11C]CIMBI-36) as their radioligand. We undertook sensitivity analyses excluding data from Erritzoe et al., 2022, as this was the only study to use an agonist tracer and to have groups unmatched for age (although age was included as a covariate in analyses) [46]. With this study excluded, 5-HT_2A_R binding was significantly lower in MDD in temporal cortex (*g* = –0.45 [–0.81, –0.09], *p*_*uncorrected*_ = 0.015, *p*_*corrected*_ = 0.045; I^2^ = 39%, *p* = 0.15; *N* = 6) (see Fig. 4D for this forest plot) and there were no significant changes to results in other regions (*p* > 0.10, data not shown).

**Fig. 5:**
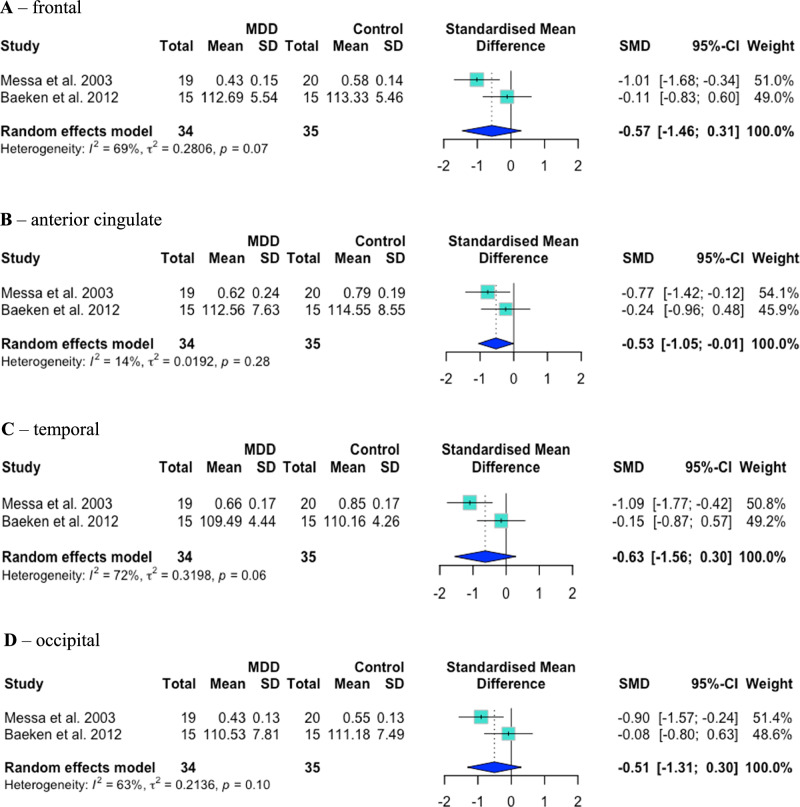
Case–control PET/SPECT studies: exploratory forest plots. Mean 5-HT_2A_ binding was significantly lower in antidepressant-naïve MDD patients than controls in anterior cingulate cortex (**B**) only. There was no significant difference between antidepressant-naïve MDD patients and controls in frontal (**A**), temporal (**C**) or occipital (**D**) cortex.

Two case–control studies included cohorts of MDD patients who had never taken antidepressants. Messa et al. [44] found significantly lower 5-HT_2A_R binding in antidepressant-naïve MDD than controls in frontal, anterior cingulate, temporal and occipital cortex [44]. Meanwhile, Baeken et al. [53] found non-significantly lower binding in antidepressant-naïve MDD in frontal, ventral prefrontal, orbitofrontal, anterior cingulate, temporal and occipital cortex, and non-significantly higher binding in dorsal prefrontal and parietal cortex [53]. In exploratory analyses, 5-HT_2A_R binding was significantly lower in antidepressant-naïve MDD patients than controls in anterior cingulate cortex (*g* = –0.53 [–1.05, –0.01], *p*_*uncorrected*_ = 0.047, *p*_*corrected*_ = 0.047, I^2^ = 14%, *p* = 0.28; *N* = 2) only. There were no significant differences in frontal (*g* = –0.57 [–1.46, 0.31], *p*_*uncorrected*_ = 0.20, *p*_*corrected*_ = 0.20; I^2^ = 69%, *p* = 0.07; *N* = 2), temporal (*g* = –0.63 [–1.56, 0.30], *p*_*uncorrected*_ = 0.18, *p*_*corrected*_ = 0.18; I^2^ = 72%, *p* = 0.06; *N* = 2) or occipital (*g* = –0.51 [–1.31, 0.30], *p*_*uncorrected*_ = 0.22, *p*_*corrected*_ = 0.36; I^2^ = 83%, *p* = 0.10; *N* = 2) cortex. See Fig. 5 for these four forest plots.

#### Meta-analysis of group differences in binding variance

The variability of 5-HT_2A_R binding was not significantly different between MDD patients and controls in any region. See Supplementary Figure 7 for lnVR, lnCVR, VR, CVR and associated *p* values. See Supplementary Figure 8 for seven lnVR and seven lnCVR forest plots.

#### Meta-regression of mean baseline HDRS score against binding effect size

Data from Attar-Lévy et al. [45] were excluded because the authors reported using the 26-item questionnaire; accordingly, the mean baseline HDRS score in the patient group was more than two SDs above the population mean (z = 2.79).

**Fig. 6:**
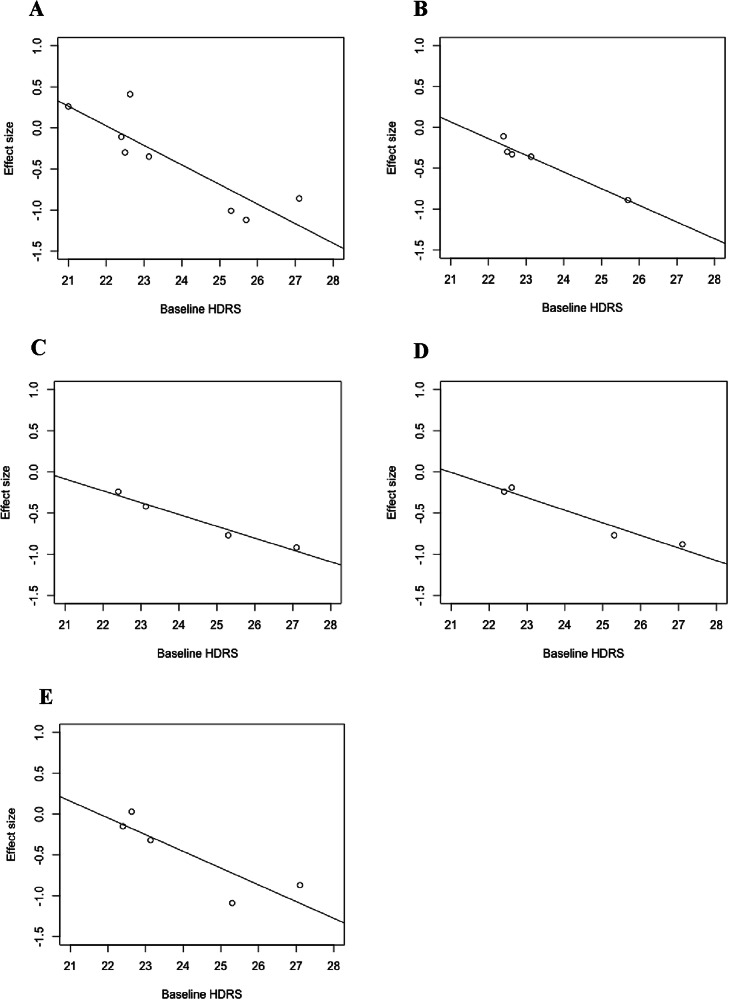
Case–control PET/SPECT studies: meta-regressions of mean baseline HDRS score against study-level regional effect size. A significant negative correlation was seen in frontal (**A**), prefrontal (**B**), cingulate (**C**) and anterior cingulate (**D**) cortex. A non-significant (*p* = 0.057) negative correlation was seen in temporal cortex (excluding Erritzoe et al. 2022) (**E**).

There was a significant negative correlation between mean baseline HDRS score and 5-HT_2A_R binding effect size in MDD patients in frontal (adjusted R^2^ = 0.70, F(df regression 1, df residual 6) = 17.6, *p*_*uncorrected*_ = 0.0057, *p*_*corrected*_ = 0.006; number of studies, *N* = 8), prefrontal (R^2^ = 0.92, F(1,3) = 48.83, *p*_*uncorrected*_ = 0.006, *p*_*corrected*_ = 0.006; *N* = 5), cingulate (R^2^ = 0.95, F(1,2) = 61.8, *p*_*uncorrected*_ = 0.016, *p*_*corrected*_ = 0.025; *N* = 4) and anterior cingulate (R^2^ = 0.93, F(1,2) = 38.25, *p*_*uncorrected*_ = 0.025, *p*_*corrected*_ = 0.025; *N* = 4) cortex. No significant correlation was seen in temporal cortex (excluding data from Erritzoe et al. 2022 [46]) (R^2^ = 0.67, F(1,3) = 9.06, *p* = 0.057; *N* = 5). See Fig. 6 for these five regressions.

#### Publication bias

It was not possible to assess funnel plot asymmetry visually in any region due to the small number of included studies (see Supplementary Figure 9 for all seven funnel plots). However, Egger’s test was non-significant in all regions; that is, frontal (*p* = 0.42), prefrontal (*p* = 0.18), cingulate (*p* = 0.72), anterior cingulate (*p* = 0.13), temporal (*p* = 0.46), occipital (*p* = 0.42) and parietal (*p* = 0.40) cortex.

#### Study-level risk of bias

We found four studies to be of good quality, five studies to be of fair quality and two studies to be of poor quality. See Supplementary Table 3 for the full Newcastle–Ottawa Scale ratings.

### Post-mortem studies

MDD patients versus controls

#### Meta-analysis of group differences in mean binding

Three studies reported separate data for medicated and unmedicated MDD patients [58, 65, 72]. When unmedicated patient data from these three studies were used, no patient–control differences in 5-HT_2A_R binding were seen in frontal cortex (*g* = –0.16 [–0.44, 0.12], *p*_*uncorrected*_ = 0.26, *p*_*corrected*_ = 0.26; I^2^ = 0%, *p* = 0.55; *N* = 7), temporal cortex (*g* = –0.34 [–0.75, 0.08], *p*_*uncorrected*_ = 0.12, *p*_*corrected*_ = 0.23; I^2^ = 16%, *p* = 0.30; *N* = 3) or hippocampus (*g* = –0.52 [–1.05, 0.01], *p* = 0.053, *p*_*uncorrected*_ = 0.053, *p*_*corrected*_ = 0.106; I^2^ = 47%, *p* = 0.15; *N* = 3). See Supplementary Figure 10 for these three forest plots. When medicated patient data from these three studies were used, 5-HT_2A_R binding was only significantly lower in MDD in frontal cortex before correction for multiple comparisons (*g* = –0.30 [–0.59, −0.00], *p*_*uncorrected*_ = 0.0498, *p*_*corrected*_ = 0.0996; I^2^ = 0%, *p* = 0.56; *N* = 7) and there was no significant change to the results in any other region. See Supplementary Figure 11 for these three forest plots.

All meta-analysed studies used [3H]ketanserin as their radioligand.

#### Meta-analysis of group differences in binding variance

The variability of 5-HT_2A_R binding was not significantly different between MDD patients and controls in any region. See Supplementary Figure 12 for lnCVR, lnVR, CVR, VR and associated *p* values. See Supplementary Figure 13 for three lnVR and three lnCVR forest plots.

#### Publication bias

It was not possible to assess funnel plot asymmetry visually in any region due to the small number of included studies (see Supplementary Figure 14 for these three funnel plots). However, Egger’s test was non-significant in all regions; that is, frontal (*p* = 0.54), temporal (*p* = 0.74) and occipital (*p* = 0.069) cortex.

#### Study-level risk of bias

We found four studies to be of fair quality and three studies to be of poor quality. See Supplementary Table 3 for the full Newcastle–Ottawa Scale ratings.

### Post-mortem studies

Suicide victims versus controls

#### Meta-analysis of group differences in mean binding

Two studies reported separate data for medicated and unmedicated suicide victims [58, 65]. When unmedicated patient data from these two studies were used, no patient–control differences in 5-HT_2A_R binding were seen in frontal cortex (*g* = 0.22 [–0.07, 0.50], *p*_*uncorrected*_ = 0.14, *p*_*corrected*_ = 0.23; I^2^ = 61%, *p* < 0.01; *N* = 17), prefrontal cortex (*g* = 0.48 [–0.17, 1.13], *p*_*uncorrected*_ = 0.15, *p*_*corrected*_ = 0.19; I^2^ = 74%, *p* < 0.01; *N* = 7), temporal cortex (*g* = 0.04 [–0.38, 0.45], *p*_*uncorrected*_ = 0.22, *p*_*corrected*_ = 0.43; I^2^ = 53%, *p* = 0.05; *N* = 7) or hippocampus (*g* = –0.29 [–0.75, 0.17], *p*_*uncorrected*_ = 0.87, *p*_*corrected*_ = 0.87; I^2^ = 50%, *p* = 0.09; *N* = 5). See Supplementary Figure 15 for these four forest plots. There was no significant change to results in any region when medicated patient data from these two studies were used instead. See Supplementary Figure 16 for these four forest plots.

Four studies reported separate data for suicide victims who died and who did not die by violent means [58, 59, 64, 65]. In no region was 5-HT_2A_R binding significantly different between victims of either violent or non-violent suicide and controls. See Supplementary Figure 17 and 18 for these six forest plots. There was no significant difference in binding between victims of violent and non-violent suicide in frontal cortex (*p* = 0.77; number of studies, *N* = 4) (see Supplementary Figure 19 for this forest plot), and there were too few data to complete direct comparisons for other regions.

Of the studies meta-analysed, three used a 5-HT_2A_R antagonist ([3H]spiperone), eleven used an inverse agonist (2-[123I]ketanserin) and three used an agonist ([125]lysergic acid diethylamide, LSD) as their radioligand. There was no significant change to the results in any region when studies using the agonist [125I]LSD [60, 62, 68] were removed from analyses (*p* > 0.10, data not shown).

#### Meta-analysis of group differences in binding variance

The variability of 5-HT_2A_R binding was significantly higher in suicide victims than controls in frontal cortex (VR = 1.21, *p*_*uncorrected*_ = 0.0026, *p*_*corrected*_ = 0.0052), but this did not survive controlling for mean scaling (CVR = 1.10, *p*_*uncorrected*_ = 0.20, *p*_*corrected*_ = 0.40). There were no significant case–control differences in group variability in any other region. See Supplementary Figure 20 for lnCVR, lnVR, CVR, VR and associated *p* values. See Supplementary Figure 21 for four lnVR and four lnCVR forest plots.

#### Publication bias

Funnel plot inspection indicated a degree of publication bias in frontal cortex but there were too few studies for visual assessment in other regions (see Supplementary Figure 22 for these four funnel plots). Egger’s test was non-significant in all regions; that is, frontal cortex (*p* = 0.75), prefrontal cortex (*p* = 0.82), temporal cortex (*p* = 0.15) and hippocampus (*p* = 0.73).

#### Study-level risk of bias

We found three studies to be of good quality, five studies to be of fair quality and ten studies to be of poor quality. See Supplementary Table 3 for the full Newcastle–Ottawa Scale ratings.

### PET and SPECT studies

Within subjects data

Six within-subjects studies, looking at whether antidepressant treatment altered 5-HT_2A_R binding in MDD and if this was related to clinical outcome, are summarised in Supplementary Table 4. Four of these studies reported significant reductions in cortical binding post-treatment: by 18% across frontal, temporal, parietal and occipital cortex following clomipramine treatment [45]; by 8.1% across frontal, temporal, parietal and occipital cortex following desipramine treatment [75]; by 10% across various frontal, cingulate and temporal subregions following paroxetine treatment in young, but not middle-aged, adults [33]; and by 3.8% across various frontal, temporal and occipital subregions following ECT [77]. One study reported significantly increased cortical binding post-treatment: by 31% across frontal and occipital cortex following fluvoxamine treatment [76]. One study reported mixed findings post-treatment: reduced binding in right and left dorsolateral prefrontal cortex, but increased binding in left hippocampus, following high-frequency repetitive transcranial magnetic stimulation (HF-rTMS) [52]. Only one study found a significant correlation between changes in HDRS scores and changes in regional binding [52]: in right and left dorsolateral prefrontal cortex, where improvements in HDRS correlated with increased binding, and in right hippocampus, where improvements in HDRS correlated with reduced binding. Pre- and post-treatment binding data from three studies [45, 75, 76] could be included in meta-analyses below.

Supplementary Table 5 summarises three within-subjects studies looking at whether 5-HT_2A_R binding prior to antidepressant treatment predicts subsequent clinical response. No significant associations were found between baseline 5-HT_2A_R binding and antidepressant response to clomipramine [45], paroxetine [33] or HF-rTMS [52].

#### Meta-analysis of mean binding pre- versus post-treatment

5-HT_2A_R binding was not significantly different before versus after antidepressant drug treatment in the same patients in frontal (*g* = 0.78 [–0.95, 2.50], *p* = 0.38; I^2^ = 86%, *p* < 0.01; *N* = 3), temporal (*g* = 0.98 [–1.12, 3.07], *p* = 0.36; I^2^ = 88%, *p* < 0.01; *N* = 3) or occipital (*g* = 1.37 [–1.61, 4.36], *p* = 0.37; I^2^ = 91%, *p* < 0.01; *N* = 3) cortex. See Supplementary Figure 23 for these three forest plots.

## Discussion

We found that in vivo cortical 5-HT_2A_R binding was lower in unmedicated MDD patients than in healthy controls, as measured using mostly antagonist or inverse agonist tracers. Binding was significantly lower in frontal, prefrontal, cingulate, anterior cingulate and, upon sensitivity analysis, temporal cortex, with medium effect sizes (Hedges’ *g* = –0.40 to –0.57). These findings are broadly, though not entirely, consistent with previous reviews [18, 27, 28]. Discrepancies may be due to differences in review methodology. Unlike Nikolaus et al. [27, 28], we completed meta-analyses of mean difference and excluded studies of remitted patient cohorts, as significant differences in 5-HT_2A_R binding have been demonstrated between euthymic and currently depressed MDD patients [75–78]. We additionally searched multiple databases, included more studies and data, and analysed a greater number of subregions.

The results of post-mortem analyses were not consistent with in vivo findings. Frontal 5-HT_2A_R binding was only significantly lower in MDD patients than controls when medicated patient data were included and before correction for multiple comparisons, whilst the results of all other post-mortem analyses were negative. We also found no significant effect of suicide on 5-HT_2A_R binding. There are several possible methodological reasons for these results. First, not all studies matched cases and controls for post-mortem delay (the interval between time of death and sample processing). Second, there were between-study differences in how samples were stored: brains were variably stored whole (e.g., [55]), as cortical regions (e.g., [69]) and as slices (e.g., [60, 62, 70, 71]); storage temperatures ranged from –80 °C (e.g., [54, 58, 65, 66, 69, 70, 72]) to –40 °C (e.g., [55]); and storage times were seldom reported. Third, there were between-study differences in assay parameters: most studies homogenised brain tissue but some used sectioned slides (e.g., [60–62, 66, 67, 70, 71]); incubation temperatures ranged from 21 °C (e.g., [62]) to 37 °C (e.g., [56–59, 63, 65, 68, 72]); and incubation times ranged from 15 min (e.g., [58, 65]) to 180 min (e.g., [63, 64, 69]). Fourth, we must consider the pharmacological profile of the radioligand. All post-mortem MDD studies used [3H]ketanserin, which has classically been considered a 5-HT_2A_R antagonist [79], although there is recent evidence that it may act as a partial agonist [80]. Studies in suicide victims used either [3H]ketanserin, the antagonist [3H]spiperone or the agonist [125I]LSD and their different profiles may account for some of the between-study variance in binding values. Similarly, the fact that PET and SPECT MDD studies used antagonist, inverse agonist and agonist tracers might, at least in part, explain why in vivo findings were not supported by the results of post-mortem MDD analyses, as we will discuss in more detail later. Fifth, some studies employed a saturation binding assay (e.g., [54, 56, 58, 64, 65, 68, 69])—an approach generally favoured for estimating maximal binding—whereas others studied receptor binding at a fixed concentration of radioligand (e.g., [55, 57, 60–62, 66, 70]). Finally, most post-mortem studies compared group means and SEMs, whereas meta-analysis using Hedges’ *g* requires first converting study-level SEMs to SDs. As ${SD}={SEM}\times \sqrt{n}$, and sample sizes ranged from seven to 73 subjects per group, SD was always several times larger than SEM, which reduced the likelihood of significant study-level observations being significant at the meta-analytical level. There are also sources of biological variability within the post-mortem literature. Suicide victims had a wide range of neuropsychiatric diagnoses, including MDD, bipolar affective disorder, schizophrenia, schizoaffective disorder, personality disorder and Parkinson’s disease (e.g., [57, 63, 66]). Also, whilst all patients in included PET and SPECT studies had been antidepressant-free for at least a week, in several post-mortem MDD (e.g., [66, 71, 72]) and suicide (e.g., [57, 59, 64, 66, 68]) studies, patients were still taking antidepressants or other psychotropics at the time of death. These important limitations impact the robustness of our post-mortem analyses. The rest of our discussion will therefore focus on insights from the PET and SPECT literature.

When do alterations in cortical 5-HT_2A_R binding occur in MDD? Lower in vivo 5-HT_2A_R binding appears to result from the effects of chronic depressive illness. Baeken et al. [53] found that 5-HT_2A_R binding in frontal, dorsolateral prefrontal and anterior cingulate cortex was significantly lower in treatment-resistant MDD than both first episode MDD and controls. Meanwhile, Schins et al. [81] reported a greater case–control difference in cortical 5-HT_2A_R binding in depressed post-myocardial infarction patients who had previously experienced depression than in those who had not. This could point towards some effect of antidepressant treatment on 5-HT_2A_R binding. Accordingly, most included within-patient studies reported regional reductions in 5-HT_2A_R binding following antidepressant treatment (and whilst accompanying meta-analyses did not reach significance, they only included three studies and heterogeneity was high). Crucially, however, studies examining 5-HT_2A_R binding in never-medicated MDD also found reduced binding relative to controls, in frontal, anterior cingulate, temporal, occipital and hippocampal regions [44, 51]. When data were combined in exploratory meta-analyses, binding was significantly lower in MDD in anterior cingulate cortex, where our primary analyses found the largest binding effect size (*g* = –0.57). This suggests that, in at least some cortical areas, case–control differences in mean binding are somewhat independent of medication effects. This idea is supported by our finding that baseline HDRS scores correlated with 5-HT_2A_R binding in frontal, prefrontal, cingulate and anterior cingulate cortex, after all patients had been off antidepressants for at least a week. (Notably, the only study to include patients who had been antidepressant-free for under two weeks found no significant difference in binding between those who had been off antidepressants for less than three weeks versus more than three weeks [47]). We therefore conclude that cortical 5-HT_2A_R binding, as measured using antagonist and inverse agonist tracers, is reduced during the course of MDD—partly, but not entirely, due to the effects of antidepressant medication.

Why is cortical 5-HT_2A_R binding reduced in depression? Based on the findings of their own [18 F]setoperone studies [49, 75, 77], Yatham and colleagues proposed that cortical 5-HT_2A_Rs are downregulated as a compensatory mechanism in MDD. They suggested that endogenous 5-HT_2A_R downregulation is sufficient to induce remission from depression in some individuals, whereas treatment is required to further reduce receptor density below a critical threshold in others [49]. This idea was also put forward to explain how ECT induces response in treatment-resistant MDD: by superimposing further reductions in 5-HT_2A_R binding on reductions due to antidepressant drug treatment [77]. Whilst this explanation offers a coherent account of dynamic changes to 5-HT_2A_R densities in vivo, it is at odds with a popular view that postsynaptic 5-HT receptors should *up*regulate when synaptic 5-HT concentrations are low [82], as is thought to be the case in MDD (e.g., [46]).

The ternary complex model of receptor–ligand binding provides an alternative account. The model states that G-protein coupled receptors (GPCRs) exist in coupled (active) or uncoupled (inactive) states, where the proportion of a fixed number of receptors in each state is in equilibrium, maintained by the availability of ligand [83–86]. It is said that agonists preferentially bind the active receptor, inverse agonists preferentially bind the inactive receptor and antagonists bind both equally [83–85]. Whilst this model appears to fit the activity of agonists and antagonists at the 5-HT_2A_R [87, 88], some authors have questioned whether inverse agonists even exist at 5-HT_2A_Rs in human brain. A decade ago, Nutt and colleagues highlighted that inverse agonists had, up until that point, only been characterised at mutant 5-HT_2A_Rs [89]. Recent post-mortem studies appear to have addressed this knowledge gap, however, finding that altanserin, pimavanserin, ritanserin, volinanserin, eplivanserin and nelotanserin all act as inverse agonists at 5-HT_2A_Rs in healthy human brain [80, 90]. Of note, one post-mortem study using the agonist [125I]LSD, inverse agonist [18 F]altanserin and antagonist [3H]MDL100907 recently reported higher, lower and unchanged 5-HT_2A_R binding, respectively, in the same unmedicated schizophrenia patients compared to controls [91].

Thus, lower binding of inverse agonist tracers to 5-HT_2A_Rs in MDD may reflect a reduction in the density of inactive 5-HT_2A_Rs. If there were no change to the overall number of receptors, this would signify a complementary increase in the density of active 5-HT_2A_Rs, relative to controls. It would follow that further reductions in 5-HT_2A_R binding after antidepressant drug treatment and ECT represent further decreases and increases, respectively, in the densities of inactive and active 5-HT_2A_Rs. This model could account for Erritzoe and colleagues’ observation of significantly higher cortical 5-HT_2A_R binding in MDD patients than controls: their agonist tracer, [11C]CIMBI-36, may have been preferentially bound to active 5-HT_2A_Rs. However, application of the ternary complex model here relies on the assumption that the total number of (both active and inactive) receptors is stable [86], which is unlikely to always be the case. Factors such as receptor mutation, trafficking and phosphorylation may affect receptor numbers, as well as the proportion of GPCRs in active and inactive states [92]. Importantly, we have noted that antagonist tracers may not distinguish between active and inactive GPCRs [87, 91]. Yet, most studies, including those of Yatham and colleagues, employed antagonist tracers and reported lower 5-HT_2A_R binding in MDD than controls. Whilst lower binding might reflect a reduction in the density of all active and inactive 5-HT_2A_Rs in MDD, it could alternatively be that some 5-HT_2A_R “antagonists” preferentially bind inactive 5-HT_2A_Rs. Recall that altanserin and other 5-HT_2A_R ligands previously thought to be antagonists have recently been redescribed as inverse agonists [80, 90, 93, 94]. In summary, lower 5-HT_2A_R binding in MDD could reflect a reduced density of all 5-HT_2A_Rs and/or an increase in the proportion of active relative to inactive receptors; however, both these interpretations involve important assumptions or caveats.

Interestingly, several studies have found 5-HT_2A_R binding to be higher in individuals with personality risk factors for depression than in healthy controls [18, 95]. This has been best demonstrated in neuroticism [96–98], where the neurotic traits vulnerability [99], negativism [100] and pessimism [34] positively correlate with 5-HT_2A_R binding. All of these studies report similar results despite employing various agonist [98], antagonist [18, 34] and inverse agonist [96, 97, 99, 100] tracers, challenging the applicability of the ternary complex model to 5-HT_2A_R dynamics here. We therefore suggest that higher 5-HT_2A_R binding in neuroticism reflects higher densities of both active and inactive receptors, relative to controls. This may occur as an adaptation against environmental risk factors for MDD, possibly via reduced 5-HT release.

We acknowledge four main limitations of our review of the case–control PET and SPECT literature. First, we included studies regardless of psychotropic drug use and screening. Washout durations were too highly variable to standardise; however, all patients were off antidepressants and antipsychotics at the time of scanning. The most commonly continued psychotropics—benzodiazepines—may affect 5-HT_2A_R density; however, they likely increase receptor binding [101, 102], which would not have contributed to the patient–control differences we report. Second, we did not weight subregions according to size. Whilst such weighting would offset the greater impact of noise in smaller regions, it would be inappropriate to ascribe all larger regions more statistical influence in the context of MDD. Our approach is consistent with that of a prior meta-analysis of 5-HT receptors in MDD [103]. Third, some of our calculations were underpowered. There were too few data to correlate 5-HT_2A_R binding with antidepressant or psychotropic washout durations, and whilst we did not find any significant evidence of publication bias, we included fewer studies than is recommended to visually inspect funnel plots or run Egger’s test reliably [104]. Although there were sufficient data to run some additional analyses and regressions, more data are required to confirm or refute associations of borderline significance, such as the correlation between temporal cortical binding and baseline HDRS score. Finally, studies may have used different versions of the HDRS questionnaire. Some studies clearly reported using the 17- [47, 50, 52, 53] or 21-item [49] versions, which should both produce the same mathematical total and are therefore comparable [42]. Whilst not explicitly stated, we assume that Sheline et al. [51] used 17-item scale scores, as was the case for its parent study [50]; that Erritzoe et al. [46] used the 17-item questionnaire, as this is the version they reference [105]; that Meyer et al. [48] used the 21-item questionnaire, as this is the version they reference [106]; and that Messa et al. [44] used the 17- or 21-item scale, because of the required cut-off score given for study inclusion (at least 18 points), but we cannot be certain.

### Conclusion and future directions

We found that in vivo cortical 5-HT_2A_R binding was significantly lower in unmedicated MDD patients than control subjects, as measured using mostly antagonist or inverse agonist tracers. This effect was seen in frontal, prefrontal, cingulate, anterior cingulate and, upon sensitivity analysis, temporal cortex (Hedges’ *g* = –0.40 to –0.57). Meanwhile, study-level binding effect size correlated with mean HDRS score at baseline in frontal and cingulate regions. Some authors reported lower regional binding in antidepressant-naïve MDD patients than controls, and exploratory meta-analyses found that binding was lower in these patients in anterior cingulate cortex. The results of within-patient studies indicated that antidepressants further reduce 5-HT_2A_R binding in MDD. These findings were not supported by analyses of the corresponding post-mortem literature, which were almost entirely negative. We suggest that this is at least in part due to methodological and biological differences across studies.

Further longitudinal studies, reporting complete medication histories, are required to fully disentangle psychotropic effects from illness effects at the 5-HT_2A_R. Ideally, these studies would recruit individuals at high risk of MDD prior to the onset of clinical depression and would follow them up throughout illness and treatment. We also encourage PET or SPECT investigations of 5-HT_2A_R binding within clinical trials of classical psychedelics for MDD and other mental health conditions, such as addictions and anxiety disorders. These studies should specifically investigate relationships between baseline 5-HT_2A_ binding, changes in 5-HT_2A_ binding with treatment and treatment outcomes. Finally, ambitious studies should seek to scan the same subjects with two or more radiotracers with different pharmacodynamic properties, to compare the differential effects of agonist, antagonist and inverse agonist tracers in PET and SPECT studies of 5-HT_2A_Rs in MDD.

## Supplementary information

Supplementary material

PRISMA checklist
