# Supplementary material for: Cortical 5-HT2A receptors in depression and suicide: a systematic review and meta-analysis of in vivo and post-mortem imaging studies
Source: Mol Psychiatry. 2025 Oct 6;30(12):6045–62. doi: 10.1038/s41380-025-03233-4 (PMC12602341; doi:10.1038/s41380-025-03233-4)
Supplement: Supplementary file 1 — Supplementary material [file 41380_2025_3233_MOESM1_ESM.docx]

PubMed (via NLM) – 1974–present, non-human subject and non-English language articles removed

*(((PET OR positron emission tomography OR SPET OR SPECT OR single photon emission tomography OR single photon emission computed tomography)[MeSH Terms]) AND ((depress* OR depressive disorder OR major depressive disorder OR MDD OR major depressive episode OR MDE)[MeSH Terms])) AND ((5-HT2* OR serotonin2* OR serotonin-2* OR serotonin 2*)[MeSH Terms])*

EMBASE (via Ovid) – 1974–present, non-human subject and non-English language articles removed

*((PET or positron emission tomography or SPET or SPECT or single photon emission tomography or single photon emission computed tomography) and (depress* or depressive disorder or major depressive disorder or MDD or major depressive episode or MDE) and (5-HT2* or serotonin2* or serotonin-2* or serotonin 2*)*

APA PsycINFO (via Ovid) – 1806–present, non-human subject and non-English language articles removed

*((PET or positron emission tomography or SPET or SPECT or single photon emission tomography or single photon emission computed tomography) and (depress* or depressive disorder or major depressive disorder or MDD or major depressive episode or MDE) and (5-HT2* or serotonin2* or serotonin-2* or serotonin 2*)*

Web of Science – no limits applied

*((ALL=((PET OR positron emission tomography OR SPET OR SPECT OR single photon emission tomography OR single photon emission computed tomography))) AND ALL=((depress* OR depressive disorder OR major depressive disorder OR MDD OR major depressive episode OR MDE))) AND ALL=((5-HT2* OR serotonin2* OR serotonin-2*))*

*Supplementary Figure 1*. **Search strategies for identifying PET/SPECT studies of 5-HT_2A_ binding in MDD.**

**A**

EMBASE (via Ovid) – 1974–present, human subjects only, English language only, pre-print articles removed

*((post mortem or post-mortem or autops* or autoradiograph* or auto-radiograph*) and (5-HT2* or serotonin2* or serotonin-2*) and (depress* or depressive disorder or major depressive disorder or MDD or major depressive episode or MDE))*

PubMed (via NLM) – 1869–present, human subjects only, English language only, pre-print articles removed

*(((post mortem) OR (post-mortem) OR (autops*) OR (autoradiograph*) OR (auto-radiograph*)[MeSH Terms]) AND ((5-HT2*) OR (5-HT(2*)) OR (2*R) OR (serotonin2*) OR (serotonin-2*) OR (serotonin 2*)[MeSH Terms])) AND ((depress*) OR (depressive disorder) OR (major depressive disorder) OR (MDD) OR (major depressive episode) OR (MDE)[MeSH Terms])*

APA PsycINFO (via Ovid) – 1806–present, human subjects only, English language only

*((post mortem or post-mortem or autops* or autoradiograph* or auto-radiograph*) and (5-HT2* or serotonin2* or serotonin-2*) and (depress* or depressive disorder or major depressive disorder or MDD or major depressive episode or MDE))*

Web of Science – no limits applied

*((ALL=((post mortem) OR (post-mortem) OR (autops*) OR (autoradiograph*) OR (auto-radiograph*))) AND ALL=((5-HT2*) OR (serotonin2*) OR (serotonin-2*))) AND ALL=((depress*) OR (depressive disorder) OR (major depressive disorder) OR (MDD) OR (major depressive episode) OR (MDE))*

**B**

EMBASE (via Ovid) – 1974–present, human subjects only, English language only, pre-print articles removed

*((post mortem or post-mortem or autops* or autoradiograph* or auto-radiograph*) and (5-HT2* or serotonin2* or serotonin-2*) and (suicid* or parasuicid* or self harm* or self-harm*))*

PubMed (via NLM) – 1869–present, human subjects only, English language only, pre-print articles removed

*(((post mortem) OR (post-mortem) OR (autops*) OR (autoradiograph*) OR (auto-radiograph*)[MeSH Terms]) AND ((5-HT2*) OR (5-HT(2*)) OR (2*R) OR (serotonin2*) OR (serotonin-2*) OR (serotonin 2*)[MeSH Terms])) AND ((suicid* or parasuicid* or self harm* or self-harm*)[MeSH Terms])*

APA PsycINFO (via Ovid) – 1806–present, human subjects only, English language only

*((post mortem or post-mortem or autops* or autoradiograph* or auto-radiograph*) and (5-HT2* or serotonin2* or serotonin-2*) and (suicid* or parasuicid* or self harm* or self-harm*))*

Web of Science – no limits applied

*((ALL=((post mortem) OR (post-mortem) OR (autops*) OR (autoradiograph*) OR (auto-radiograph*))) AND ALL=((((5-HT2*) OR (serotonin2*) OR (serotonin-2*)))) AND ALL=((suicid*) or (parasuicid*) or (self harm*) or (self-harm*))*

*Supplementary Figure 2*. **Search strategies for identifying post-mortem studies of 5-HT_2A_ binding in A) MDD and B) suicide.**

**A**

| Inclusion criteria | Exclusion criteria |
| --- | --- |
| - Quantitative autoradiographic study of cortical 5-HT_2A_ binding in depression - Using a radioligand with satisfactory 5-HT_2A_ affinity and selectivity - Mean regional binding values reported, with standard deviation (SD) or standard error of the mean (SEM) - For patients: history of MDE or MDD; depressed at death - For controls: no known history of MDE or MDD; not depressed at death - All dates of publication - Human subjects of all ages | - For patients: history of bipolarity; comorbid psychiatric or neurological illness; significant physical illness; euthymic at time of scanning - Controls: any psychiatric, neurological or significant physical illness - Non-human subjects - Non-English language - Review article or inappropriate study design - Non peer-reviewed publication (e.g. conference abstract, letter to editor, preprint) - Duplicate hit (in current or previous search) |

**B**

| Inclusion criteria | Exclusion criteria |
| --- | --- |
| - Quantitative autoradiographic study of cortical 5-HT_2A_ binding in suicide - Using a radioligand with satisfactory 5-HT_2A_ affinity and selectivity - Mean regional binding values reported, with standard deviation (SD) or standard error of the mean (SEM) - For patients: death by suicide; may or may not have psychiatric diagnosis - For controls: non-suicidal cause of death; no known psychiatric diagnosis - All dates of publication - Human subjects of all ages | - For patients: comorbid neurological illness; significant physical illness - Controls: any psychiatric, neurological or significant physical illness - Non-human subjects - Non-English language - Review article or inappropriate study design - Non peer-reviewed publication (e.g. conference abstract, letter to editor, preprint) - Duplicate hit (in current or previous search) |

*Supplementary Figure 3*. **Inclusion and exclusion criteria post-mortem studies of 5-HT_2A_ binding in A) MDD and B) suicide.**

**296** records identified

**59** duplicate records removed before screening

**237** records screened

**212** records excluded

**25** full-text reports sought

**1** full-text not available

**24** reports assessed for eligibility

**15** reports excluded

- *8* Inappropriate patient population (mixed diagnosis group / diagnosis not stated)
- *2* Provisional results (Letter to Editors / conference abstract)
- *2* Duplicate
- *1* Review article
- *1* Inappropriate control group
- *1* Incorrect outcomes reported

**Identification of studies via databases and registers**

**Identification**

**Screening**

**9** case–control studies included

Including Rosel et al., 1998; Rosel et al., 2000

**Included**

*Supplementary Figure 4*. **PRISMA flow-chart for finding, screening, excluding and including post-mortem studies of 5-HT_2A_ binding in MDD.**

**167** records screened

**130** records excluded

**37** full-text reports sought

**2** full-text not available

**35** reports assessed for eligibility

**Identification of studies via databases and registers**

**Identification**

**Screening**

**15** reports excluded

- *5* Duplicate
- *3* Review article
- *3* Inappropriate control group
- *3* Inappropriate patient population (included non-suicides)
- *1* Provisional results (Letter to Editors)

**20** case–control studies included

Including Rosel et al., 1998; Rosel et al., 2000; Muguruza et al., 2013

**Included**

*Supplementary Figure 5*. **PRISMA flow-chart for finding, screening, excluding and including post-mortem studies of 5-HT_2A_ binding in suicide.**

**224** records identified

**57** duplicate records removed before screening

PET/SPECT studies: MDD versus controls

Frontal cortex: PFC, dlPFC, OFC, SFG, MFG, IFG, GR

Prefrontal cortex: PFC, dlPFC, OFC

Cingulate cortex: ACC, pgCC, sgCC, CG

Temporal cortex: aTC, pTC, lTC, STG, ITG, FFG

Parietal cortex: SPC

Post-mortem studies: MDD versus controls

Frontal cortex: dlPFC

Temporal cortex: HC

Post-mortem studies: completed suicide versus controls

Frontal cortex: PFC, SFG, MFG, IFG, GR, precentral gyrus, orbital gyri

Temporal cortex: STG, MTG, ITG, FFG, HC

Hippocampus: CA, DG, subiculum

aTC, anterior temporal cortex; CA, cornu ammonis; CG, cingulate gyrus; DG, dentate gyrus; dlPFC, dorso-/dorsolateral prefrontal cortex; FFG, fusiform gyrus; GR, gyrus rectus; IFG, inferior frontal cortex/gyrus; ITG, inferior temporal cortex/gyrus; lTC, lateral temporal cortex; MFG, medial frontal cortex/gyrus; MTG, medial temporal cortex/gyrus; OFC, orbitofrontal cortex; PFC, prefrontal cortex; pgCC, pregenual cingulate cortex; pTC, posterior temporal cortex; SFG, superior frontal cortex/superior frontal gyrus; sgCC, subgenual cingulate cortex; sgPFC, subgenual prefrontal cortex; SPC, superior parietal cortex; STG, superior temporal cortex/gyrus.

*Supplementary Figure 6.* **Strategy for combining subregions to give ‘pooled’ regions for meta-analysis.** Where available, binding values for whole regions were included in meta-analyses over ‘pooled’ values; thus, regional meta-analyses that included whole regional data only are not listed above.

| **Region** | **lnVR** | **lnCVR** | **VR** | **CVR** |
| --- | --- | --- | --- | --- |
| Frontal cortex | –0.087 (uncorrected *p* = 0.34, corrected *p* = 0.34) | 0.033 (uncorrected *p* = 0.71, corrected *p* = 0.71) | 0.92 | 1.03 |
| Prefrontal cortex | –0.15 (uncorrected *p* = 0.28, corrected *p* = 0.34) | –0.048 (uncorrected *p* = 0.68, corrected *p* = 0.71) | 0.86 | 0.95 |
| Cingulate cortex | –0.21 (uncorrected *p* = 0.20, corrected *p* = 0.40) | –0.0074 (uncorrected *p* = 0.96, corrected *p* = 0.96) | 0.81 | 0.99 |
| Anterior cingulate cortex | –0.11 (uncorrected *p* = 0.53, corrected *p* = 0.53) | 0.068 (uncorrected *p* = 0.68, corrected *p* = 0.96) | 0.90 | 1.07 |
| Temporal cortex | –0.18 (*p* = 0.098) | –0.071 (*p* = 0.47) | 0.84 | 0.93 |
| Occipital cortex | –0.17 (*p* = 0.27) | –0.11 (*p* = 0.46) | 0.84 | 0.90 |
| Parietal cortex | –0.12 (*p* = 0.39) | –0.10 (*p* = 0.40) | 0.89 | 0.90 |

*Supplementary Figure 7.* **Case–control PET/SPECT studies: variability measures**. lnVR and lnCVR were backtransformed, by $e^{lnVR}$ or $e^{lnCVR}$, to give VR and CVR, respectively. There were no significant case–control differences in lnVR or lnCVR in any region.


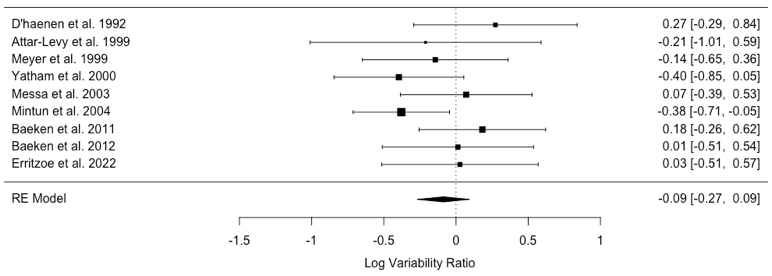

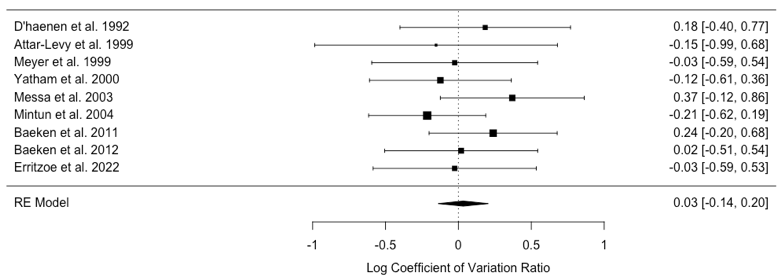

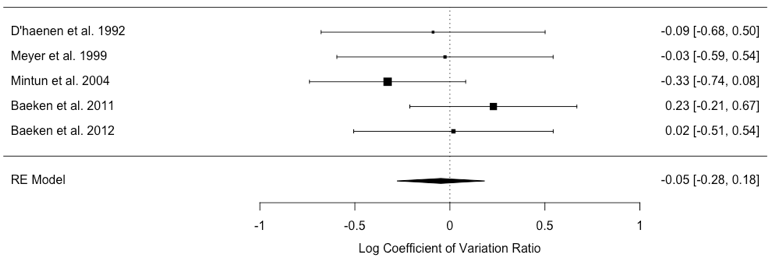

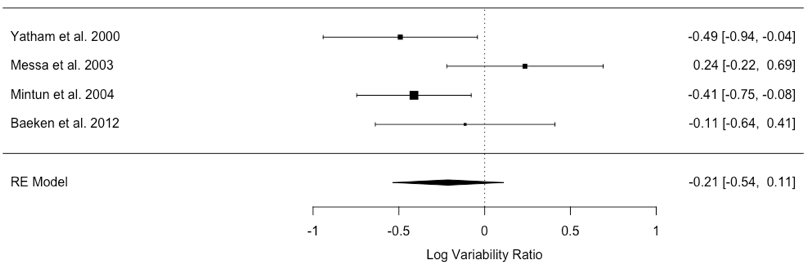

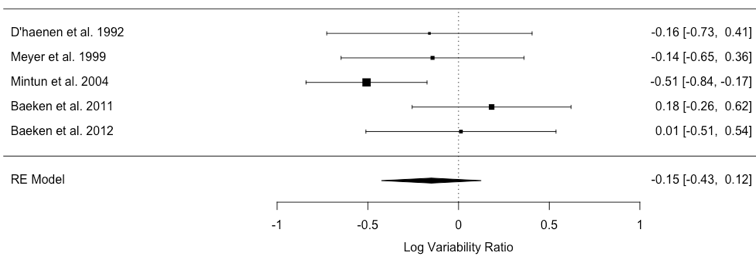

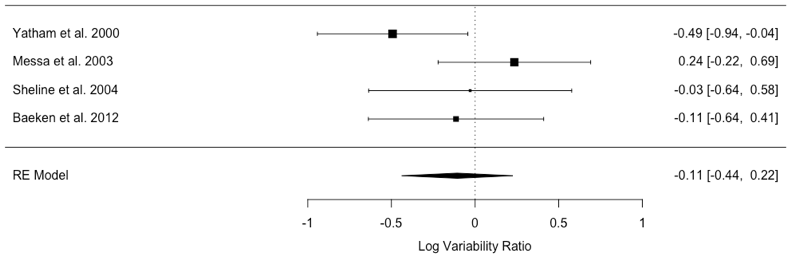

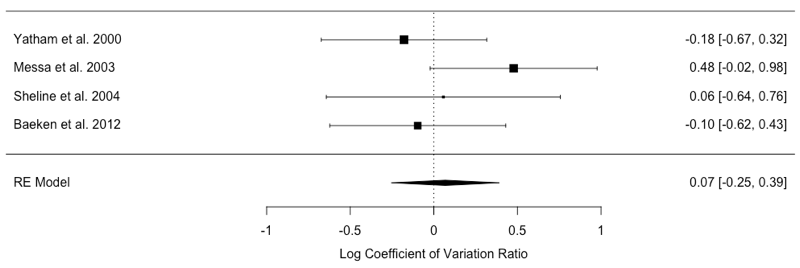

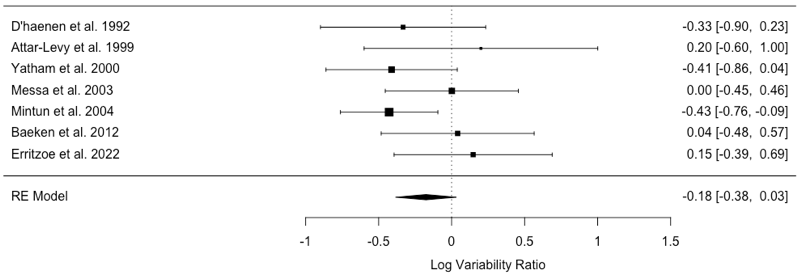

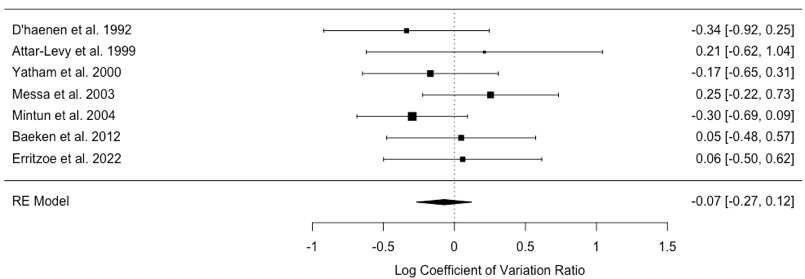

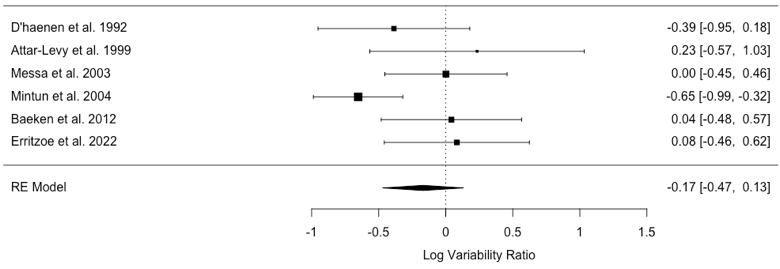

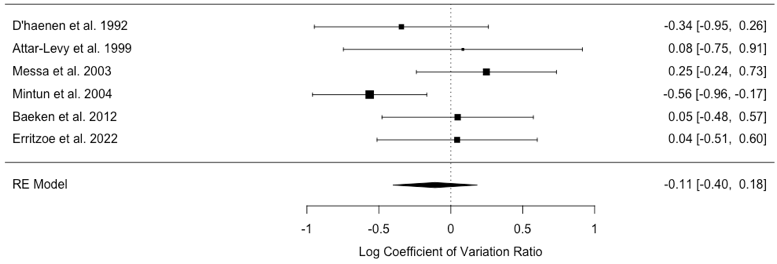

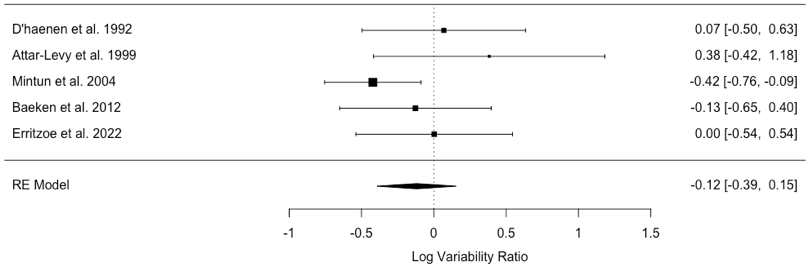

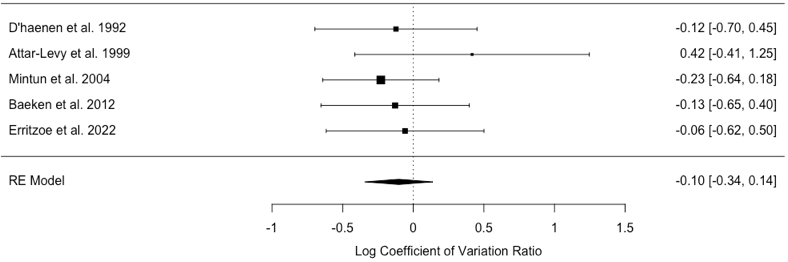

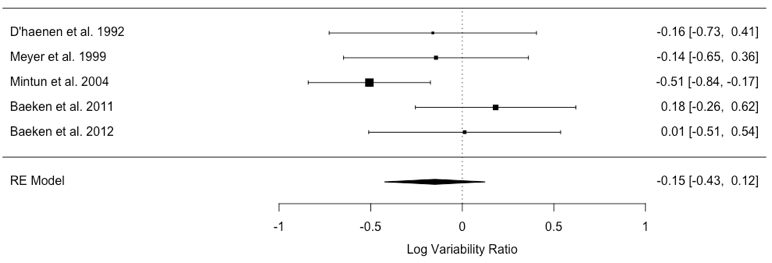


*Supplementary Figure 8.* **Case–control PET/SPECT studies: forest plots for lnVR and lnCVR.** Frontal (A), prefrontal (B), cingulate (C), anterior cingulate (D), temporal (E), occipital (F) and parietal (G) cortex. There were no significant case–control differences in lnVR or lnCVR in any region.

**A**

**B**

**C**

**D**

**E**

**F**

**G**

*Supplementary Figure 9.* **Case–control PET/SPECT studies: regional funnel plots of studies included in meta-analyses of mean difference.** Frontal (A), prefrontal (B), cingulate (C), anterior cingulate (D), temporal (E), occipital (F) and parietal (G) cortex.


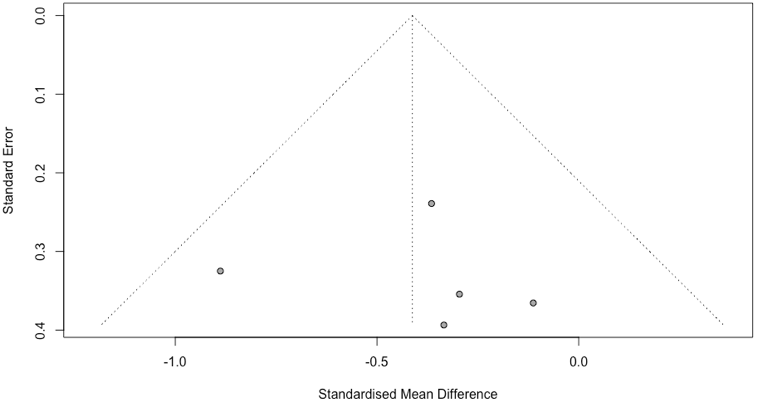

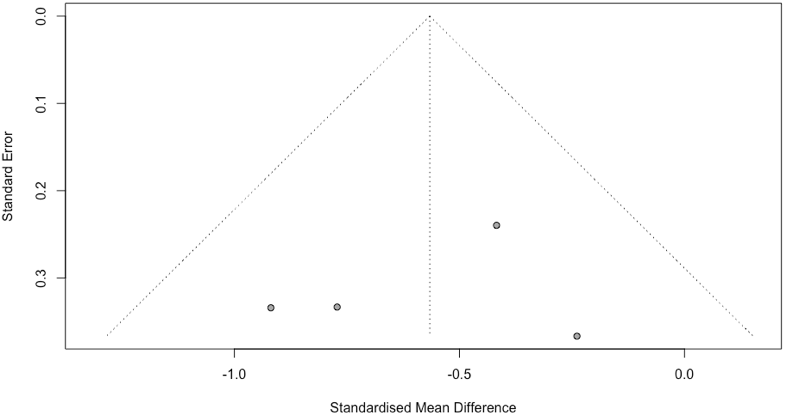

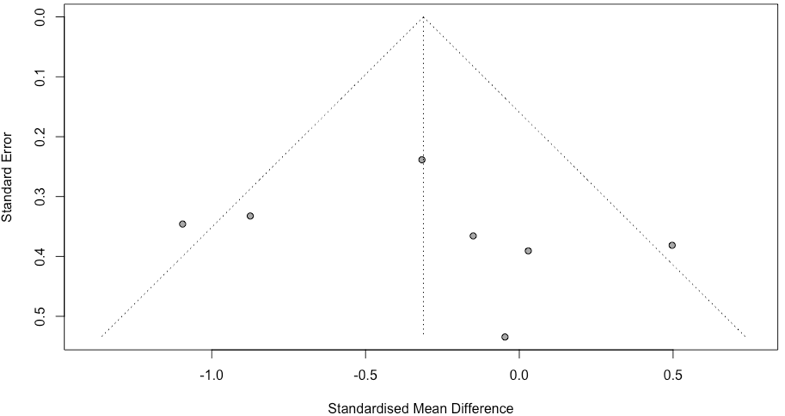

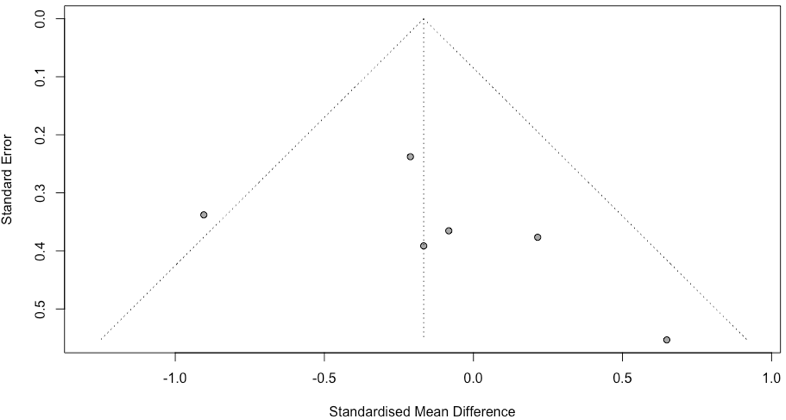

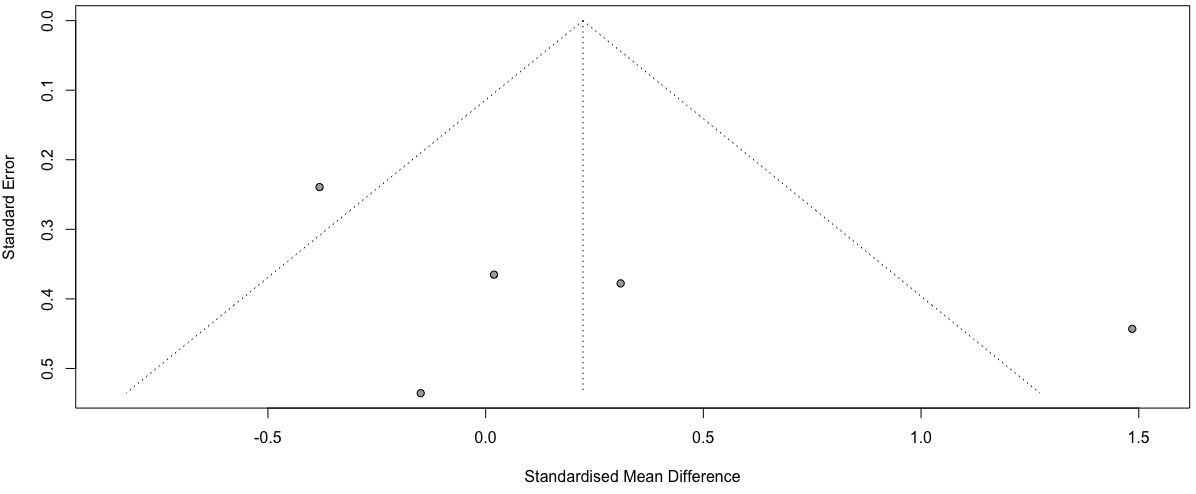

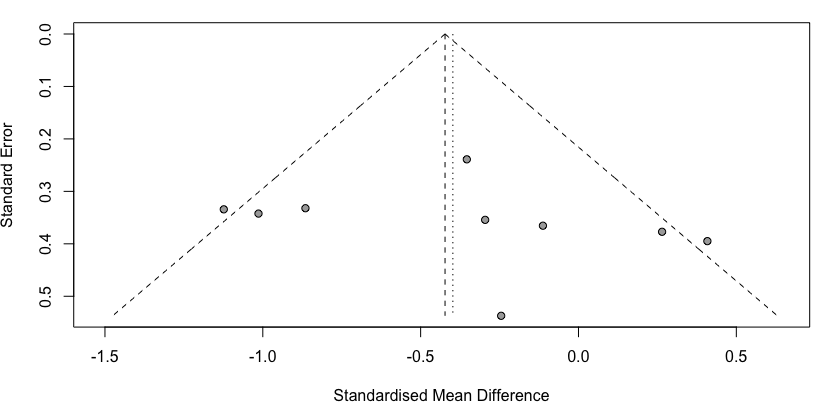


**A**

**B**

**C**

**D**

**E**

**F**

**G**


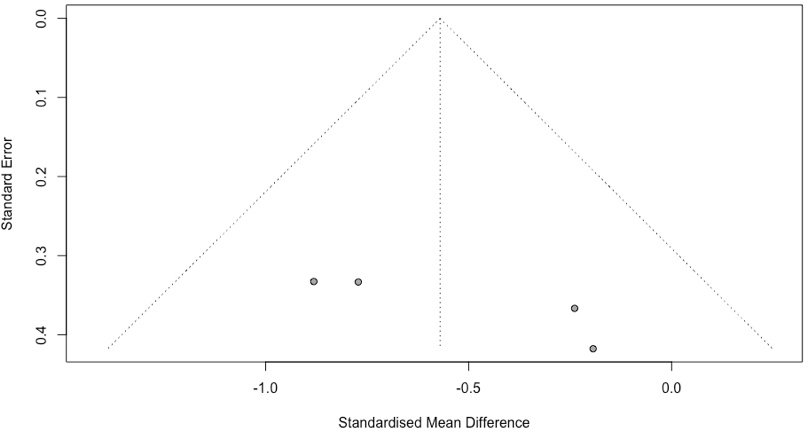


*Supplementary Figure 10*. **Post-mortem MDD studies: forest plots using unmedicated patient data.** Mean 5-HT_2A_ binding was not significantly different between MDD patients and controls in frontal cortex (A), temporal cortex (B) or hippocampus (C).


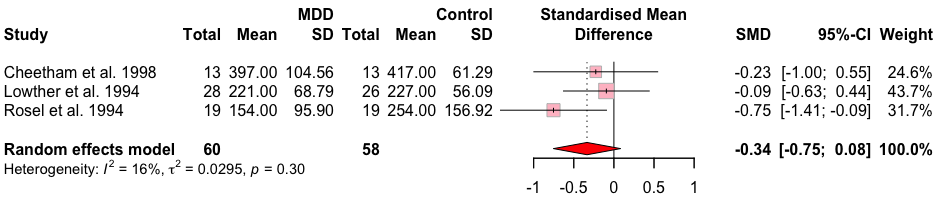

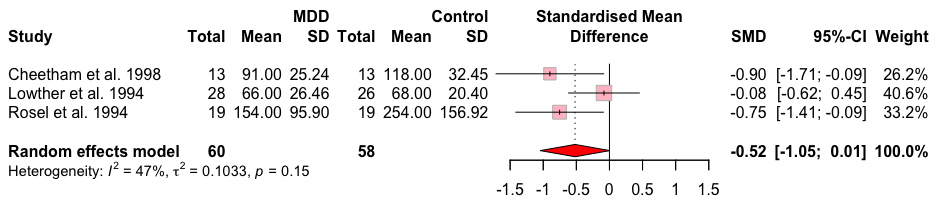

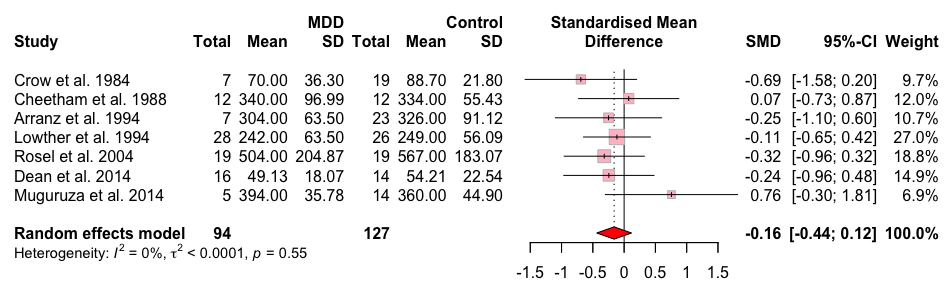


**A** – frontal

**B** – temporal

**C** – hippocampus

**A** – frontal

*Supplementary Figure 11*. **Post-mortem MDD studies: forest plots using medicated patient data.** Mean 5-HT_2A_ binding was significantly lower in MDD patients than controls in frontal cortex (A) only. There were no significant group differences in temporal cortex (B) or hippocampus (C).


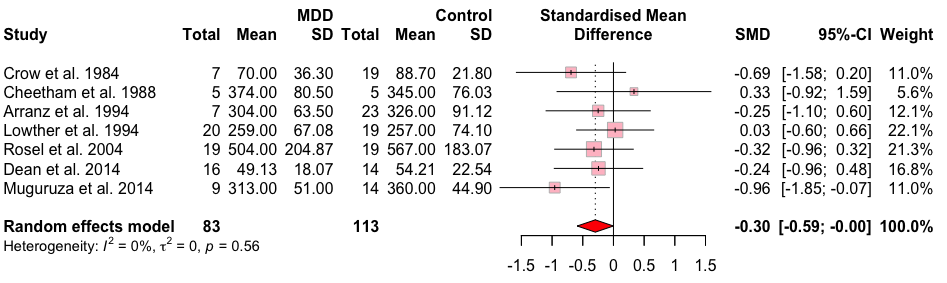

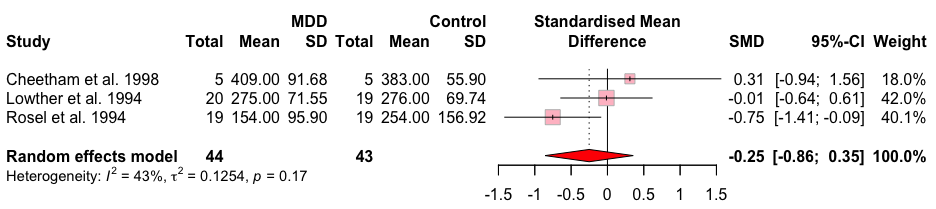

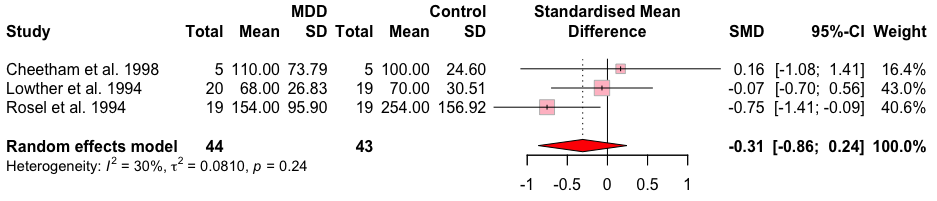


**B** – temporal

**C** – hippocampus

| **Region** | **lnVR** | **lnCVR** | **VR** | **CVR** |
| --- | --- | --- | --- | --- |
| Frontal cortex | 0.10 (*p* = 0.34) | 0.16 (*p* = 0.15) | 1.11 | 1.17 |
| Temporal cortex | 0.070 (*p* = 0.82) | 0.27 (*p* = 0.078) | 1.07 | 1.30 |
| Hippocampus | –0.14 (*p* = 0.55) | 0.15 (*p* = 0.34) | 0.87 | 1.16 |

*Supplementary Figure 12.* **Post-mortem MDD studies: variability measures (using unmedicated patient data)**. lnVR and lnCVR were backtransformed, by $e^{lnVR}$ or $e^{lnCVR}$, to give VR and CVR, respectively. There were no significant case–control differences in lnVR or lnCVR in any region.

*Supplementary Figure 13.* **Post-mortem MDD studies: forest plots for lnVR and lnCVR (using unmedicated patient data).** There were no significant case–control differences in lnVR or lnCVR in frontal cortex (A), temporal cortex (B) or hippocampus (C).


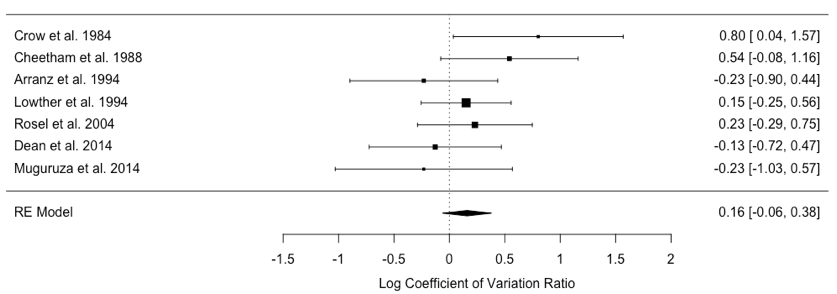

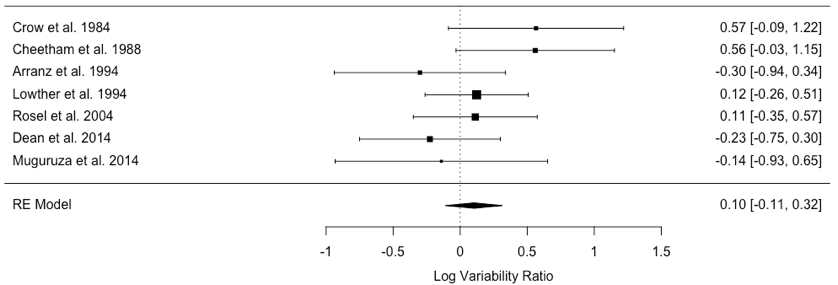

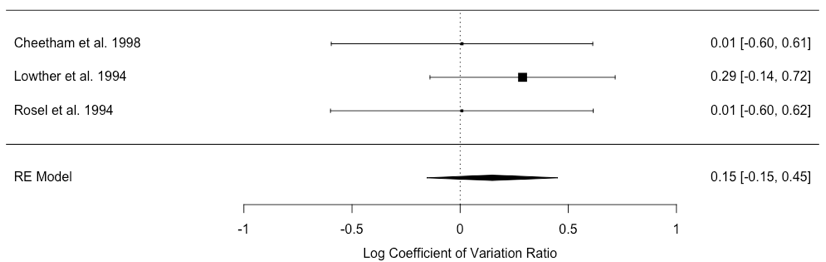

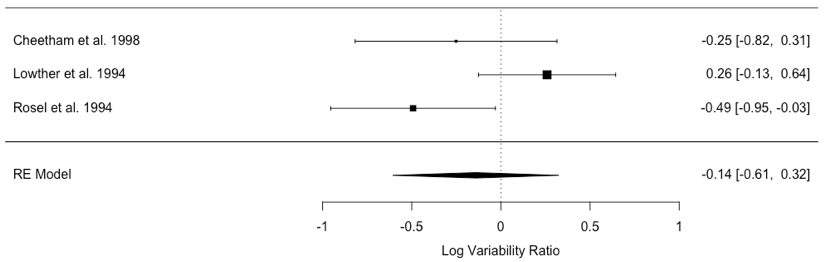

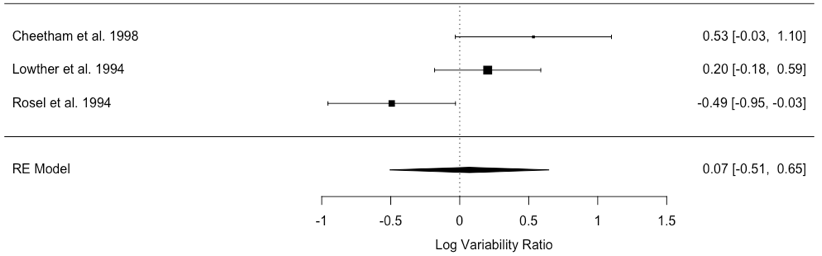

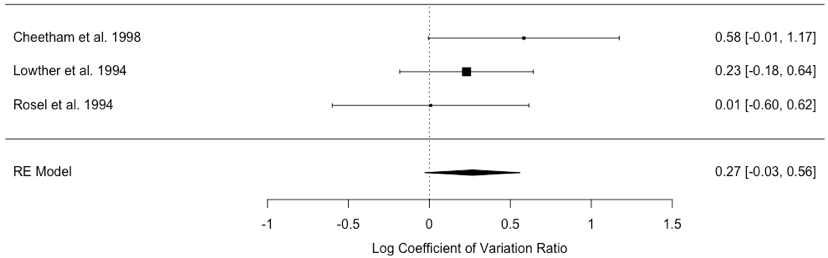


**A**

**B**

**C**

*Supplementary Figure 14.* **Post-mortem MDD studies: regional funnel plots (using unmedicated patient data).** Frontal cortex (A), temporal cortex (B), hippocampus (C).


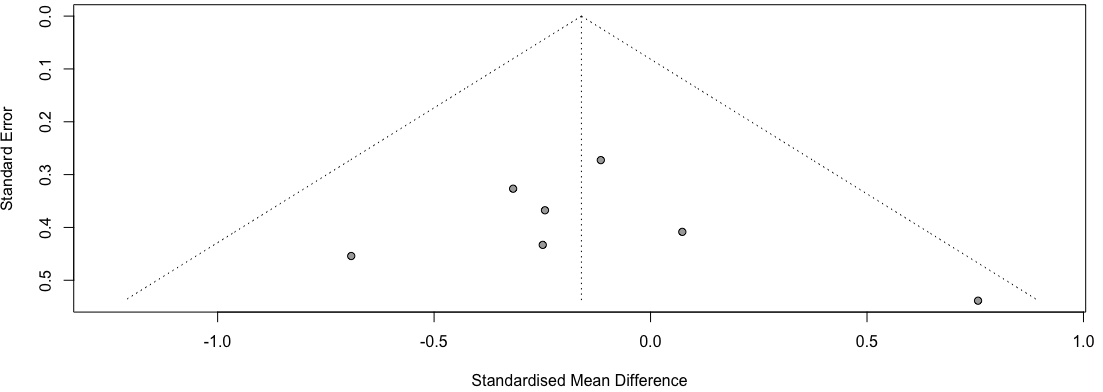

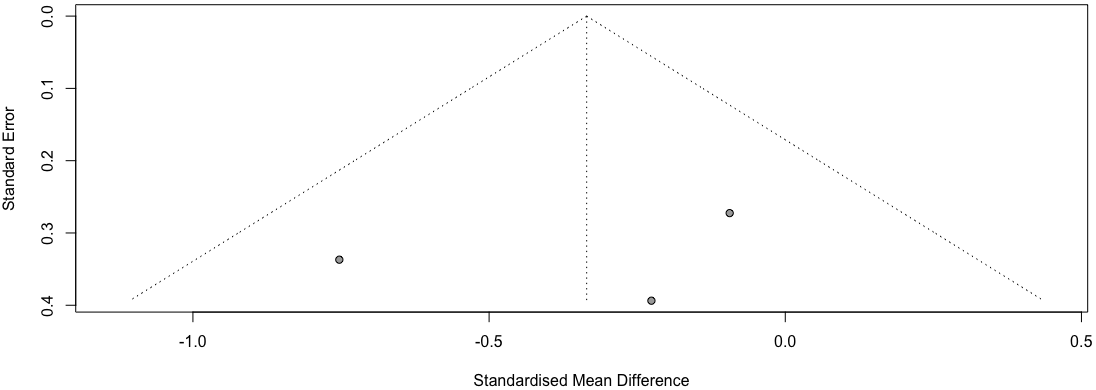

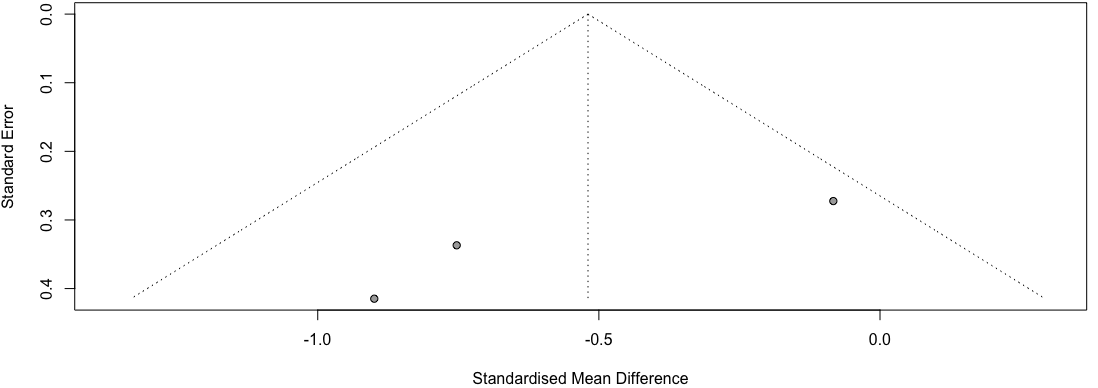


**A**

**B**

**C**

*Supplementary Figure 15*. **Post-mortem suicide studies: forest plots using unmedicated patient data.** Mean 5-HT_2A_ binding was not significantly different between suicide victims and controls in frontal cortex (A), prefrontal cortex (B), temporal cortex (C) or hippocampus (D).


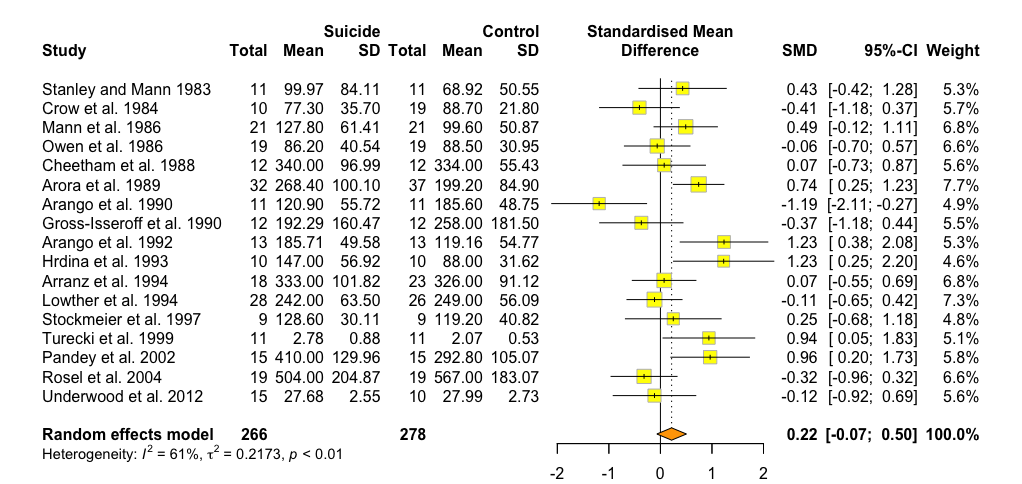

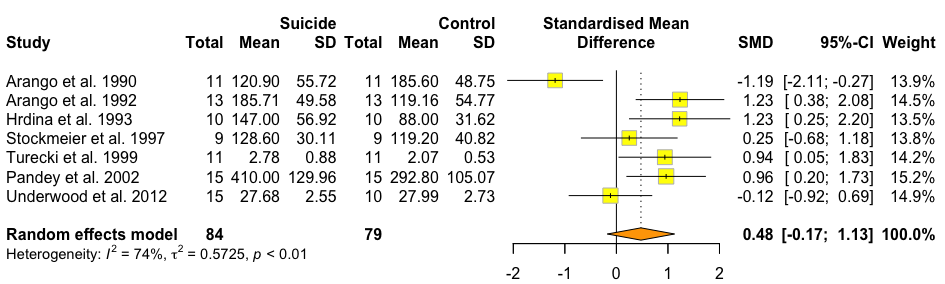

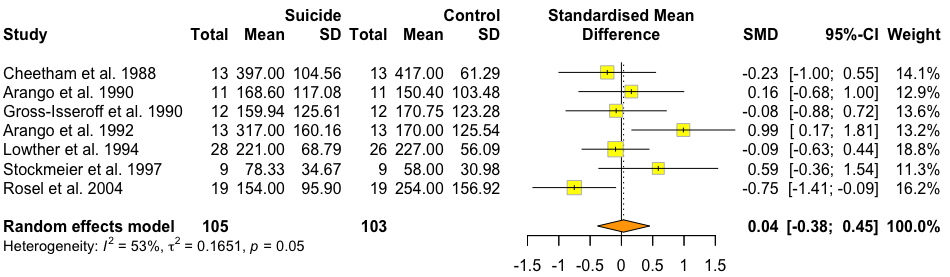

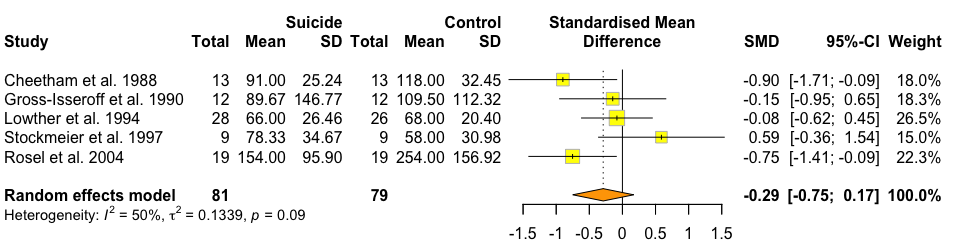


**A** – frontal

**B** – prefrontal

**C** – temporal

**D** – hippocampus

*Supplementary Figure 16*. **Post-mortem suicide studies: forest plots using medicated patient data.** Mean 5-HT_2A_ binding was not significantly different between suicide victims and controls in frontal cortex (A), temporal cortex (B) or hippocampus (C).


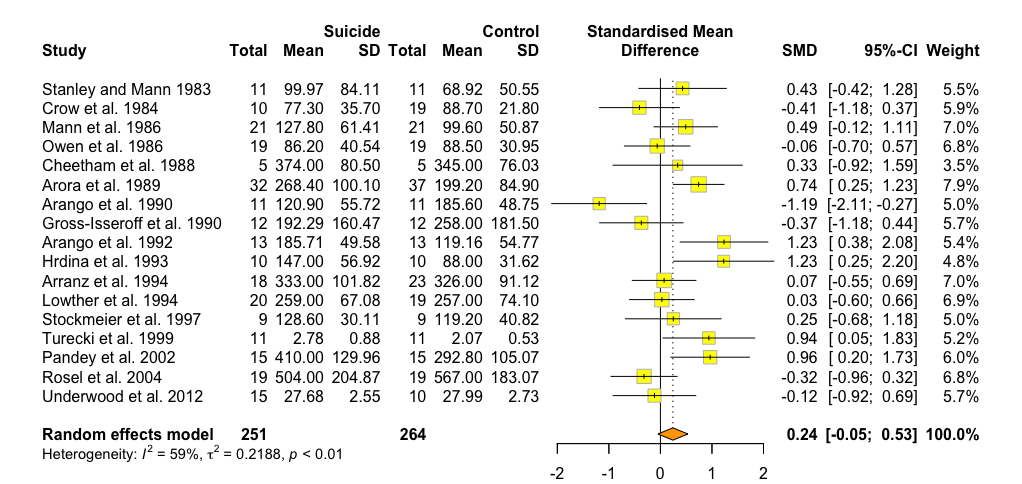

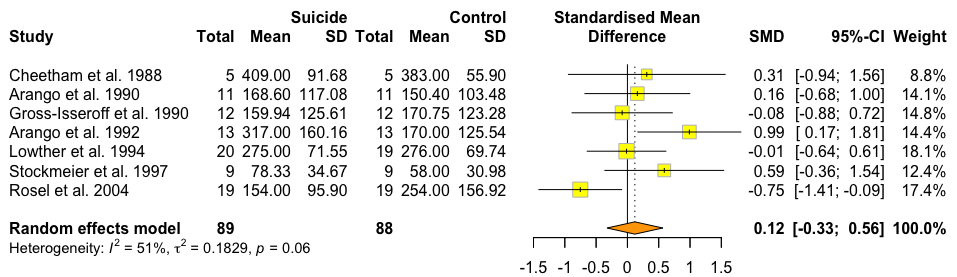

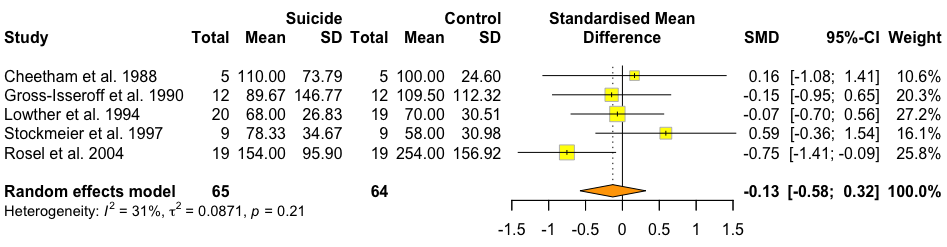


**A** – frontal

**B** – temporal

**C** – hippocampus


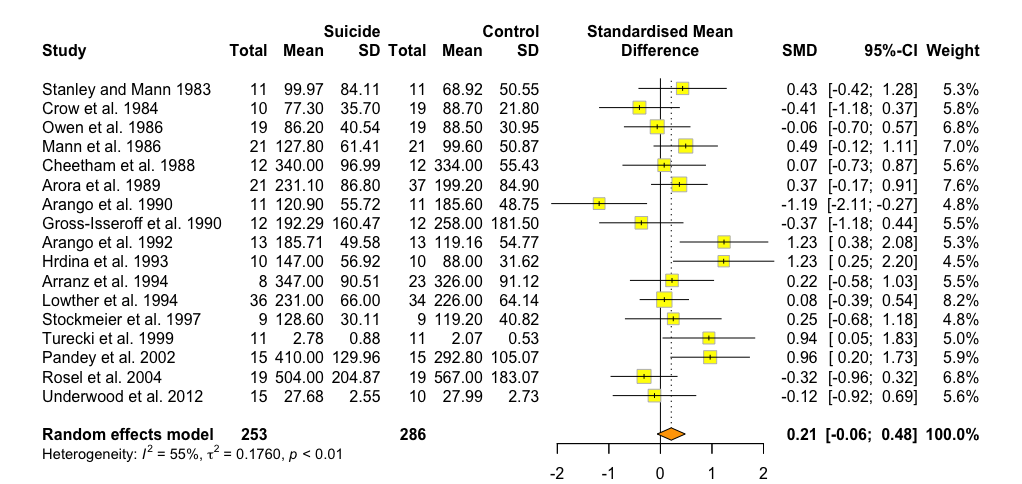

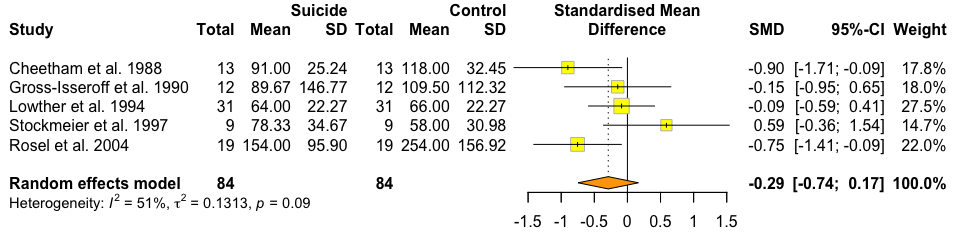


*Supplementary Figure 17*. **Post-mortem suicide studies: forest plots using violent suicide data.** 5-HT_2A_ binding was not significantly different between suicide victims and controls in frontal cortex (A), temporal cortex (B) or hippocampus (C).

**A** – frontal

**B** – temporal

**C** – hippocampus


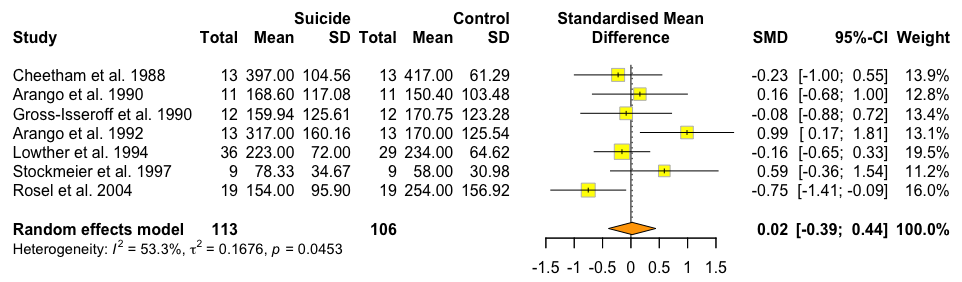


*Supplementary Figure 18*. **Post-mortem suicide studies: forest plots using non-violent suicide data.** 5-HT_2A_ binding was not significantly different between suicide victims and controls in frontal cortex (A), temporal cortex (B) or hippocampus (C).


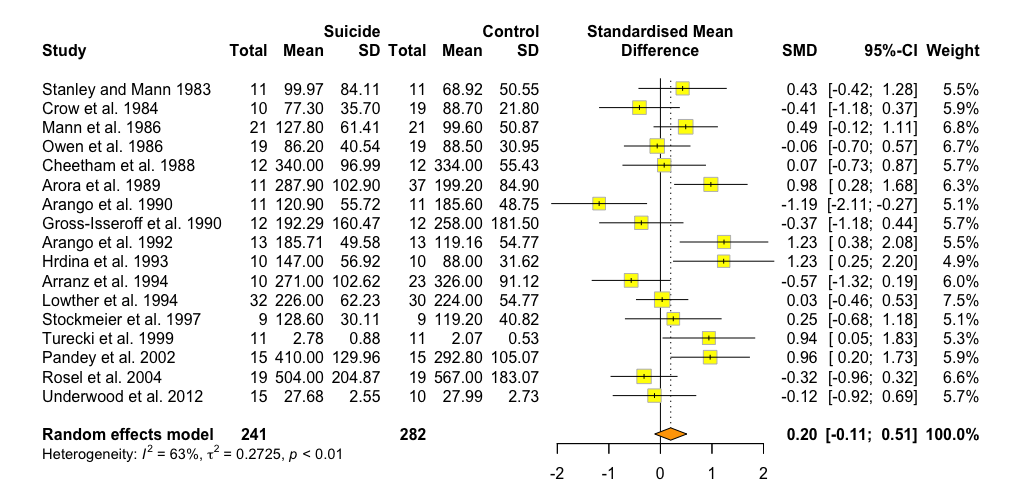

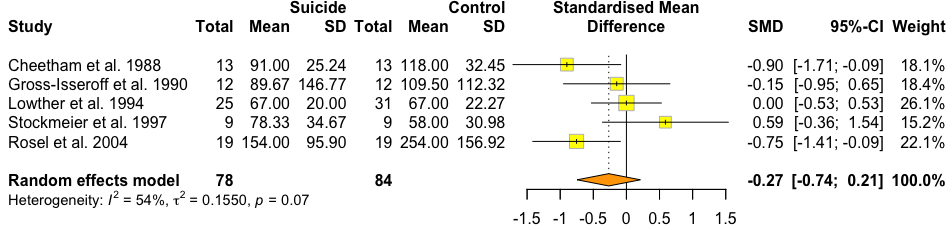


**A** – frontal

**B** – temporal

**C** – hippocampus


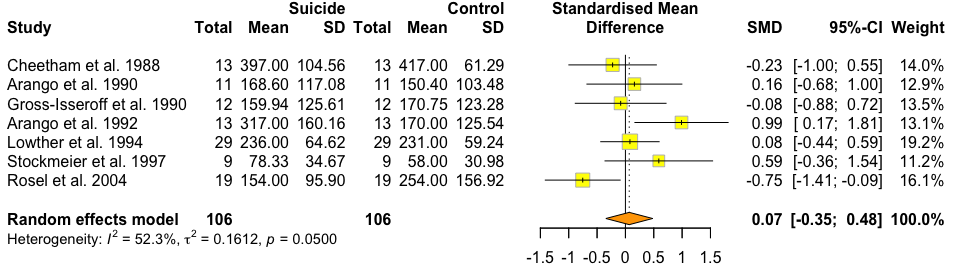


*Supplementary Figure 19*. **Post-mortem suicide studies: forest plot comparing violent and non-violent suicide data.** 5-HT_2A_ binding was not significantly different between violent and non-violent suicide victims in frontal cortex.


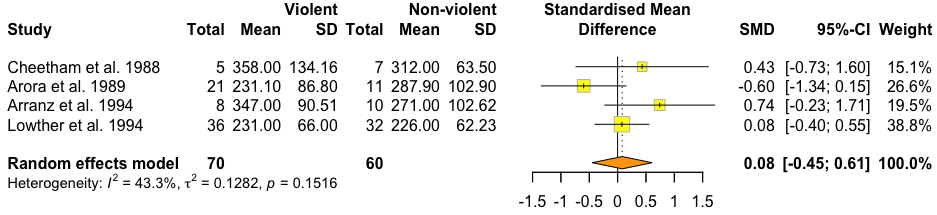


| **Region** | **lnVR** | **lnCVR** | **VR** | **CVR** |
| --- | --- | --- | --- | --- |
| Frontal cortex | 0.19 (uncorrected *p* = 0.0026, corrected *p* = 0.0052) | 0.091 (uncorrected *p* = 0.20, corrected *p* = 0.40) | 1.21 | 1.10 |
| Prefrontal cortex | 0.14 (uncorrected *p* = 0.24, corrected *p* = 0.24) | –0.050 (uncorrected *p* = 0.71, corrected *p* = 0.71) | 1.15 | 0.95 |
| Temporal cortex | 0.091 (*p* = 0.49) | 0.13 (*p* = 0.30) | 1.10 | 1.14 |
| Hippocampus | –0.027 (*p* = 0.87) | 0.13 (*p* = 0.37) | 0.97 | 1.13 |

*Supplementary Figure 20.* **Post-mortem suicide studies: variability measures (using unmedicated patient data)**. lnVR and lnCVR were backtransformed, by $e^{lnVR}$ or $e^{lnCVR}$, to give VR and CVR, respectively. lnVR was significantly greater in suicide victims than controls in frontal cortex only.

*Supplementary Figure 21.* **Post-mortem suicide studies: forest plots for lnVR and lnCVR (using unmedicated patient data).** Frontal cortex (A), prefrontal cortex (B), temporal cortex (C), hippocampus (D). lnVR was significantly greater in suicide victims than controls in frontal cortex only.


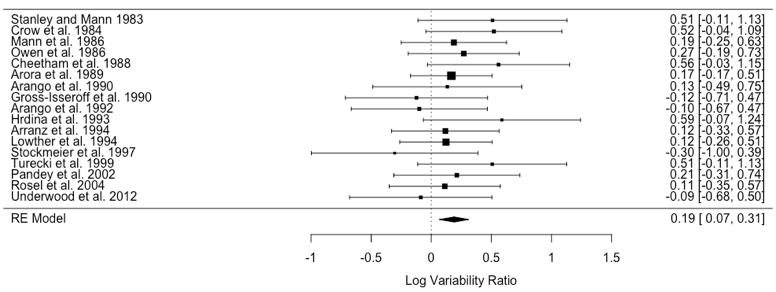

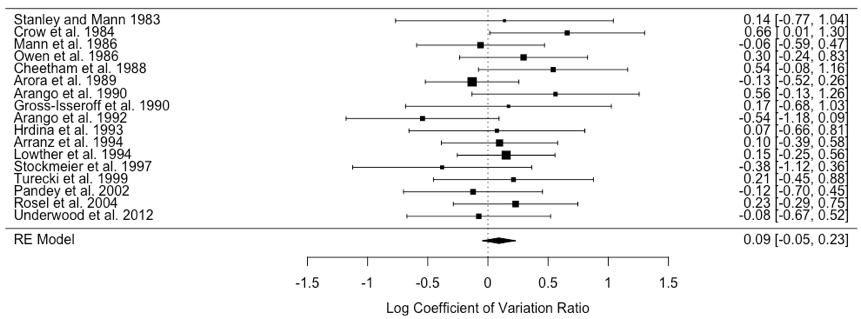

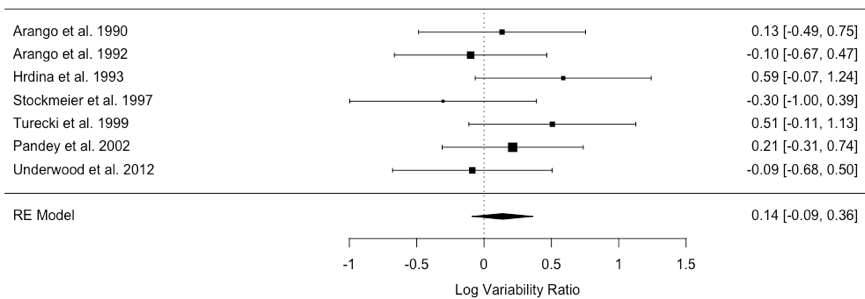

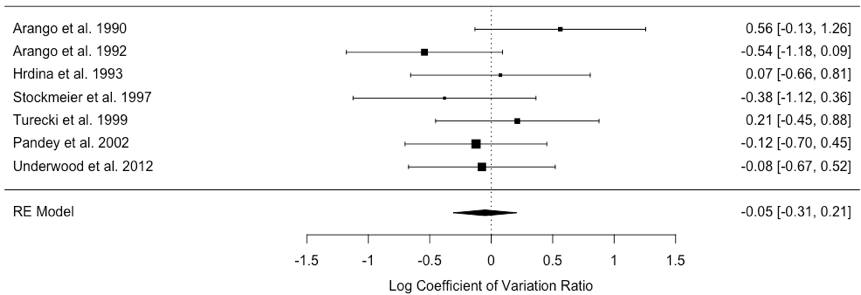

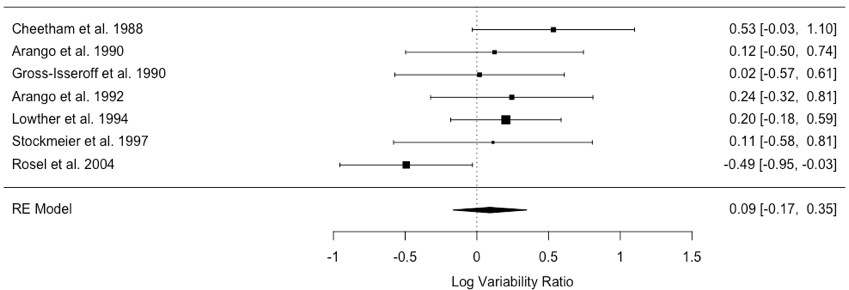

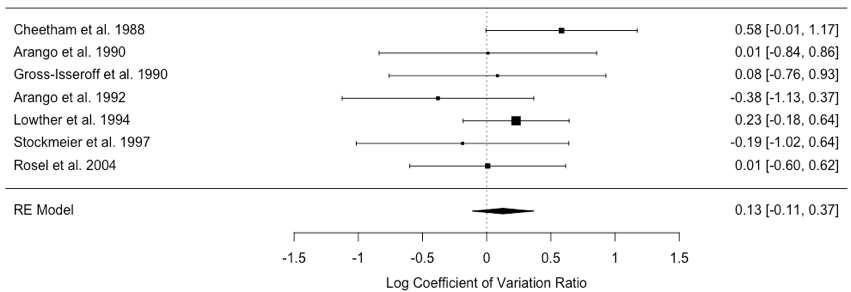

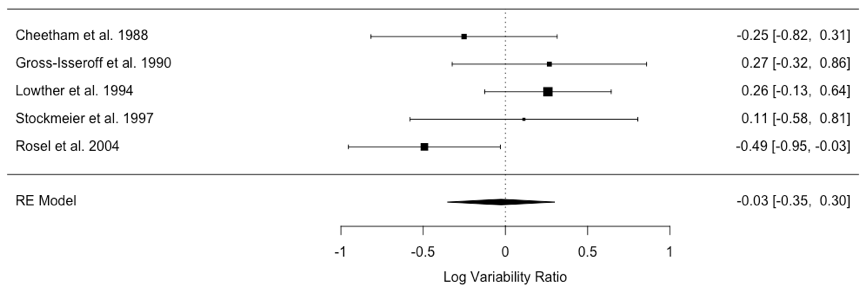

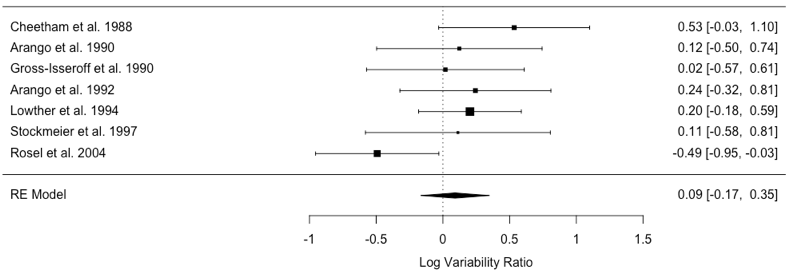


**A**

**B**

**C**

**D**

*Supplementary Figure 22.* **Post-mortem suicide studies: funnel plots (using unmedicated patient data).** Frontal cortex (A), prefrontal cortex (B), temporal cortex (C), hippocampus (D).


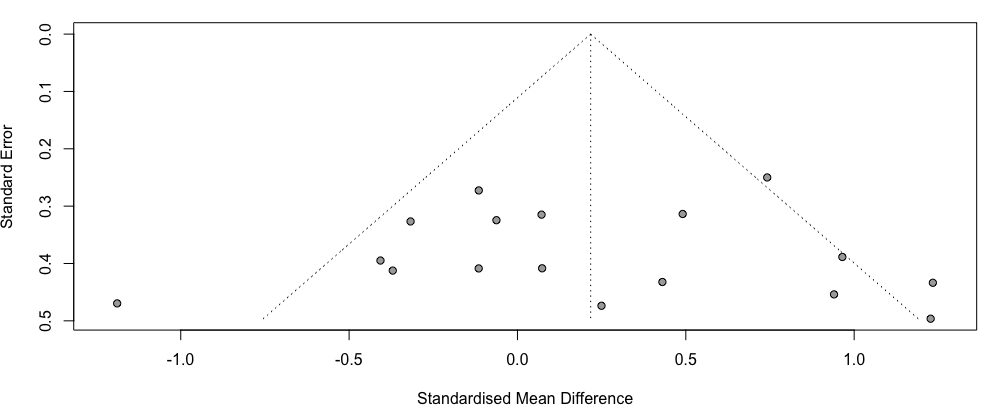

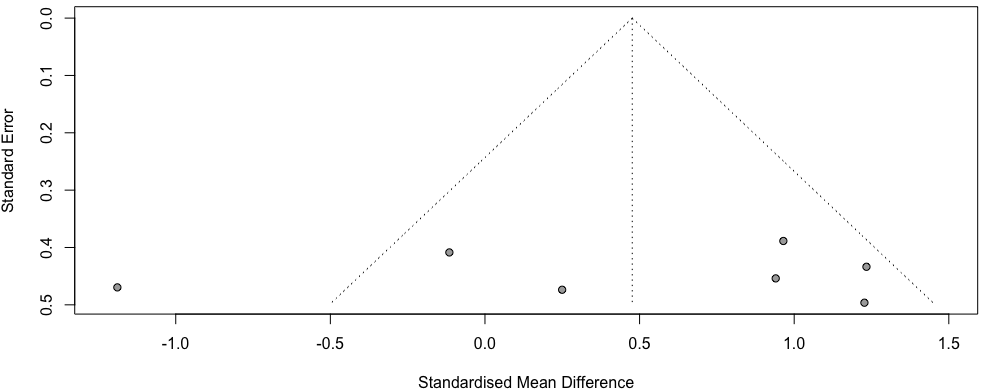

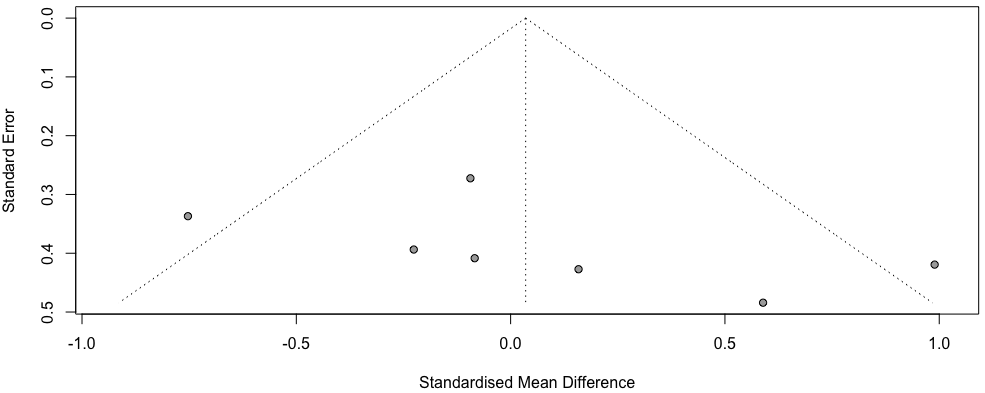

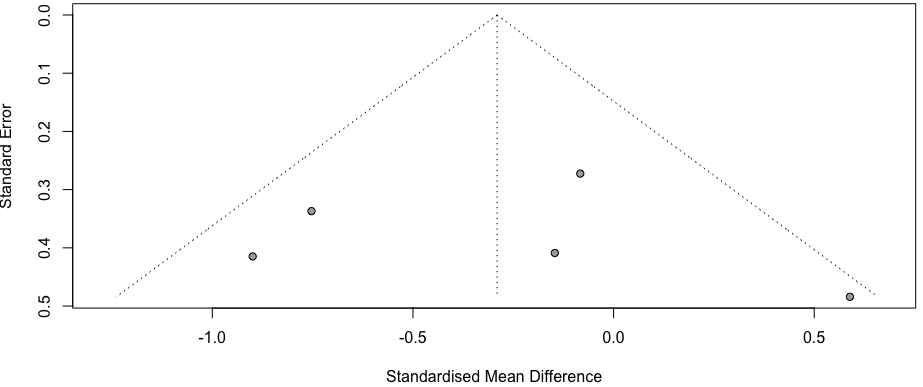


**A**

**B**

**C**

**D**

*Supplementary Figure 23.* **Within-subjects PET/SPECT data: forest plots.** Mean 5-HT_2A_ binding was not significantly different before versus after antidepressant drug treatment in the same MDD patients in frontal (A), temporal (B) or occipital (C) cortex.


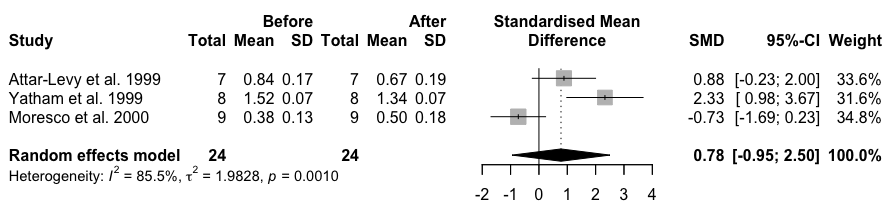

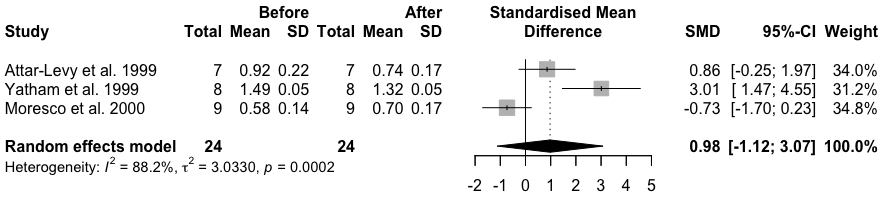

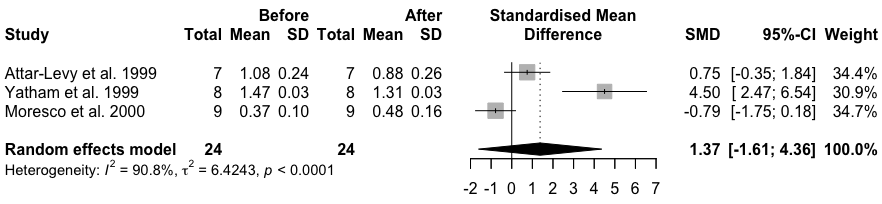


**A** – frontal

**B** – temporal

**C** – occipital

*Supplementary Table 1* – post-mortem studies: MDD versus controls

| Study | Sample | Diagnosis | Cause of death | Mean age (SD or SEM) | Age matched? | | Gender (M:F) | Gender matched? | Medication status /  toxicology details | Post-mortem delay;  Storage details;  Storage time;  Tissue processing; Radioligand used;  Incubation time and temperature | Binding in MDD relative to controls | K_d_ in MDD relative to control | Any correlation between binding value and medication status, depression severity or suicidality? |
| --- | --- | --- | --- | --- | --- | --- | --- | --- | --- | --- | --- | --- | --- |
| Crow et al. (1984) | 7 MDD suicides  (from a larger suicide cohort) | History of depressive illness (endogenous unipolar type in 3 patients) | Within larger patient cohort:  “Traumatic” means (n = 4)  BZD OD (n = 3)  AP OD (n = 1)  Analgesic OD (n = 1)  Drowning (n = 1) | 52.4 (16.3) | | Yes | 3:4 | Not reported | Not reported | Post-mortem delay not reported;  Brains removed whole and stored at –40 °C;  Storage time not reported;  Homogenised tissue;  [3H]ketanserin at 1 nM;  30 min at room temperature | (↓) in FC | Not reported | Not reported |
|  | 19 controls | Not reported | Died of “various causes in a general hospital” | 56.2 (14.6) | |  | 12:7 |  | Not reported |  |  |  | – |
| Cheetham et al. (1988) | 19 MDD suicides  **Including 13 AD-free MDD suicides** | Beskow group 1 or 2 depression, determined by psychiatrist using records and interviews  No history of schizophrenia, personality disorder, alcohol or drug abuse or epilepsy | All died by suicide –  Hanging (n = 5)  CO poisoning (n = 5)  OD (n = 5)  Wounding (n = 2)  Drowning (n = 1) | 40 (3) | | Yes | 9:4 | Yes | **In AD-free subgroup: no AD or other psychoactive drugs “recently” – no evidence on blood toxicology**  In remaining 6 patients:  AD only (n = 3)  ADs and BZD (n = 2)  BZD only (n = 1) | ND between groups for post-mortem delay;  Brains stored at –80 °C;  ND between groups for storage time;  Homogenised tissue;  [3H]ketanserin at 0.07–5.4 nM;  15 min at 37 °C | **23% ↓ in HC in AD-free subgroup vs. controls**  (↓) in TC, OC and HC in all patients vs. controls and in AD-free subgroup vs. controls  (↑) in FC in all patients vs. controls and in AD-free subgroup vs. controls | ↑ in FC, TC and OC in all patients vs. controls  ↑ in FC in AD-treated subgroup vs. controls  (↑) in FC, TC, OC and HC in AD-free subgroup vs. controls | **No effect of medication status on binding value** |
|  | 13 controls | Sudden death from non-CNS causes  No documented evidence of mental illness | Myocardial infarction (n = 16)  Fall (n = 1)  Accidental CO poisoning (n = 1)  Drowning (n = 1) | 39 (3) | |  | 9:4 |  | Not reported |  |  |  | – |
| Arranz et al. (1994) | 7 MDD suicides  (from a larger suicide cohort) | Depressive symptoms prior to death, as documented by a general practitioner or psychiatrist’s report  No history of alcohol abuse | Medical examiner stated suicide as cause of death –  Violent means (n = 4)  OD (n = 2)  CO poisoning (n = 1) | 51.5 (9.0) | | Yes | 4:3 | Yes | Evidence of psychoactive medication use | Matched for post-mortem delay;  Frontal areas were dissected and stored at –70 °C;  Storage time not reported;  Homogenised tissue;  [3H]ketanserin at 0.2–4 nmol/L;  180 min at 20 °C | (↓) in FC (BA 9, 10 and 11) | (↑) in FC | Not reported |
|  | 23 controls | No documented history of mental or CNS disorder | Ischaemic heart disease  Pulmonary disease  Accidental drowning  Road traffic accident  Accidental CO poisoning | 49.4 (3.8) | |  | 19:4 |  | No psychoactive medication use |  |  |  | – |
| Lowther et al. (1994) | **28 AD-free MDD suicides** | **Beskow group 1 or 2 depression, determined by psychiatrist using records and interviews** | **Hanging (n = 13)**  **OD (n = 6)**  **CO poisoning (n = 5)**  **Stab wound (n = 2)**  **Jump from height (n = 2)** | **45 (3)** | | **Yes** | **21:7** | **Yes** | **No ADs for at least 3 mo** | **Matched for post-mortem delay;**  **Brains were stored at –80 °C;**  **Matched for storage time;**  **Homogenised tissue;**  **[3H]ketanserin at 0.03–4 nM;**  **15 min­ at 37 °C** | **(↓) in FC, TC, and HC**  **(↑) in PC** | **(↑) in FC and PC**  **(↓) in HC**  **ND in TC in AD-free suicides vs. controls** | **Not reported** |
|  | 26 controls | Sudden death from non-CNS causes  No documented evidence of mental illness | Myocardial infarction (n = 21)  Ruptured aneurysm (n = 1)  Road traffic accident (n = 1)  Acute asthma (n = 1)  CO poisoning (n = 1)  Fall (n = 1) | 46 (3) | |  | 20:6 |  | Not reported |  |  |  | – |
|  | 20 AD-treated MDD suicides  (with different control group) | Beskow group 1 or 2 depression, determined by psychiatrist using records and interviews | OD with ADs (n = 10)  OD without ADs (n = 3)  Hanging (n = 5)  Jumping (n = 1)  CO poisoning (n = 1) | 43 (3) | | Yes (to different control group) | 10:10 | Yes (to different control group) | All taking ADs at time of death |  | (↑) in FC and PC  (↓) in TC and HC | ↑ in FC, PC, TC and HC | No effect of whether death was violent or not on binding relative to controls |
| *Rosel et al. (1998)* | ***17 AD-free MDD violent suicides*** | *DSM-III-R depressive symptoms, from joint psychiatrist–coroner interview with relative*  *No schizophrenia, personality disorder, alcohol/drug abuse or epilepsy* | *Forensic pathologist stated suicide as cause of death –*  *Hanging (n = 9)*  *Gunshot (n = 4)*  *Knife wound (n = 4)* | *42 (4.8)* | | *Yes* | *13:4* | *Yes* | ***“None … had taken any antidepressant drugs prior to death”*** | *Matched for post-mortem delay;*  *Cortical regions were dissected then stored at –80 °C;*  *Storage time not reported;*  *Homogenised tissue;*  *[3H]ketanserin at 0.3–10 nM;*  *180 min at 25 °C* | ***↓ in HC***  ***(↓) in FC***  ***(↑) in CC*** | ***↓ in HC***  ***(↓) in FC***  ***(↑) in CC*** | ***5-HT_2A_ binding was negatively correlated with 5-HTT binding in the control but not patient group***  ***No correlation found between 5-HT_2A_ binding and suicide method*** |
|  | *17 controls* | *No psychiatric or neurological disease, corroborated by joint psychiatrist–coroner interview with relative* | *Ischaemic heart disease (n = 12)*  *Road traffic accident (n = 5)* | *49 (4.0)* | |  | *15:2* |  | *Not reported* |  |  |  | *­–* |
| *Rosel et al. (2000)* | ***18 AD-free MDD violent suicides*** | ***DSM-III-R depressive symptoms, from joint psychiatrist–coroner interview with relative***  ***No schizophrenia, personality disorder, alcohol/drug abuse or epilepsy*** | ***Forensic pathologist stated suicide as cause of death –***  ***Hanging (n = 9)***  ***Gunshot (n = 4)***  ***Knife wound (n = 4)*** | ***42 (4.8)*** | | ***Yes*** | ***14:4*** | ***Yes*** | ***No ADs for at least 6 mo***  ***Never taken lithium or APs*** | ***Matched for post-mortem delay;***  ***Cortical regions were dissected then stored at –80 °C;***  ***Storage time not reported;***  ***Homogenised tissue;***  ***[3H]ketanserin at 0.3–10 nM;***  ***180 min at 25 °C*** | ***↓ in HC, associated with ↑[IP_3_]***  ***(↓) in FC, associated with (↓)[IP_3_]*** | ***↓ in HC***  ***(↓) in FC*** | ***Not reported*** |
|  | *18 controls* | *No psychiatric or neurological disease, corroborated by joint psychiatrist–coroner interview with relative* | *Ischaemic heart disease (n = 12)*  *Road traffic accident (n = 6)* | *49 (4.0)* | |  | *15:3* |  | *Not reported* |  |  |  | *–* |
| Rosel et al. (2004) | **19 AD-free MDD suicides** | **DSM-IV for MDD, from joint psychiatrist–coroner interview with relative**  **No history of schizophrenia, personality disorder, alcohol or drug abuse or epilepsy** | **Forensic pathologist stated suicide as cause of death –**  **Hanging (n = 10)**  **Gunshot (n = 5)**  **Knife wound (n = 4)** | **42 (4.8)** | | **Yes** | **15:4** | **Yes** | **No AD or AP for at least 6 mo**  **No relevant non-psychotropic medication** | **Matched for post-mortem delay;**  **Cortical regions were dissected then stored at –80 °C;**  **Storage time was up to 6 mo;**  **Homogenised tissue;**  **[3H]ketanserin at 0.3–10 nM;**  **180 min at 25 °C** | **↓ in HC, associated with ↑[IP_3_]**  **(↓) in FC, associated with (↓)[IP_3_]** | **↓ in HC** | **Not reported** |
|  | 19 controls | No psychiatric or neurological disease, corroborated by joint psychiatrist–coroner interview with relative | Ischaemic heart disease (n = 12)  Road traffic accidents (n = 7) | 49 (3.7) | |  | 15:4 |  | No relevant non-psychotropic medication |  |  |  | – |
| Dean et al. (2014) | 16 MDD | DSM-IV diagnosis, agreed by two psychiatrists and a psychologist following case note review  No history or neuropathological evidence of neurological disease | Hanging (n = 7)  Drug toxicity (n = 3)  Pneumonia (n = 2)  Drowning (n = 1)  Asphyxia (n = 1)  Cardiovascular disease (n = 1)  Deep vein thrombosis (n = 1) | 60 (3.9) | | Yes | 8:8 | Yes | All were on psychotropics at time of death –  AD(s) only (n = 12)  AP only (n = 2)  AD and APs (n = 1)  ADs and mood stabiliser (n = 1)  According to medication histories and blood toxicology | Matched for post-mortem delay;  Cut to 1 cm coronal slices then stored at –70 °C;  Storage time not reported;  Sectioned slides;  [3H]ketanserin at 10 nM;  60 min at room temperature | ↓ in BA 24 (ACC)  (↓) in BA 46 (dlPFC) | Not reported | Within larger patient cohort (including BPAD patients):  ND in binding between patients on or off ADs at time of death |
|  | 14 controls | Absence of DSM-IV diagnosis following case note review  No history or neuropathological evidence of neurological disease | Cardiovascular disease (n = 11)  Pulmonary embolism (n = 1)  Acute asthma (n = 1)  Sepsis / multi-organ failure (n = 1) | 60 (4.2) | |  | 7:7 |  | No AD, AP, mood stabiliser, BZD or anticholinergic use just prior to death  According to medication histories and blood toxicology |  |  |  | – |
| Muguruza et al. (2014) | 14 MDD  **Including 5 AD-free MDD** | DSM-IV for MDD, by case note review | 13 patients died by suicide | 53.4 (4.5) | | Yes | 5:9 | Yes | **Off ADs at death (n = 5)**  On ADs at death (n = 9)  According to blood toxicology | Matched for post-mortem delay;  dlPFC was dissected and stored at –80 °C;  Storage time not reported;  Homogenised tissue;  [3H]ketanserin at 0.03–10 nM;  60 min at 37 °C | (↓) in dlPFC | (↑) in dlPFC | **Binding was significantly lower in AD-treated MDD, but not AD-free MDD, than in control** |
|  | 14 CTL | Absence of neuropsychiatric disorder  No antemortem history of drug abuse | Mostly accidents (sudden and violent in nature) | 53.9 (4.6) | |  | 5:9 |  | Results of toxicological tests to identify AD or other drug use not reported |  |  |  | – |

↑ and ↓ denote significantly higher or lower values in MDD relative to controls, respectively. (↑) and (↓) denote non-significantly higher or lower values in MDD relative to controls, respectively. 5-HTT, serotonin transporter; ACC, anterior cingulate cortex; AD(s), antidepressant(s); AP(s), antipsychotic(s); BA, Brodmann area; BPAD, bipolar affective disorder; BZD, benzodiazepines; °C, degrees Celsius; CC, cingulate cortex; CNS, central nervous system; CO, carbon monoxide; dlPFC, dorsolateral prefrontal cortex; DSM-III(-R), Diagnostic and Statistical Manual version 3 (revised); DSM-IV, Diagnostic and Statistical Manual 4; FC, frontal cortex; HC, hippocampus; [IP_3_], concentration of inositol triphosphate (an intracellular messenger); K_d_, equilibrium dissociation constant; MDD, major depressive disorder; MDE, major depressive episode; mo, month(s); ND, no statistically significant difference; nM, nanomole; OC, occipital cortex; OD, deliberate drug overdose; OFC, orbitofrontal cortex; PC, parietal cortex; PFC, prefrontal cortex; TC, temporal cortex; TCA, tricyclic antidepressant. **Details in bold font describe findings in MDD patients who were antidepressant-free at death.** *Studies in italicised font were excluded from meta-analyses (subject groups in Rosel et al. 2004 mostly comprise subjects from Rosel et al. 1998 and 2000).*

*Supplementary Table 2* – post-mortem studies: suicide versus controls

| Study | Sample | Diagnosis | | Cause of death | Mean age (SD or SEM) | Age matched? | Gender (M:F) | Gender matched? | Medication status / toxicology details | Post-mortem delay;  Storage details;  Storage time;  Tissue processing; Radioligand used;  Incubation time and temperature | Binding in suicides relative to controls | K_d_ in suicides relative to controls | Any correlation between binding values and medication status, depression or method of suicide | |  |
| --- | --- | --- | --- | --- | --- | --- | --- | --- | --- | --- | --- | --- | --- | --- | --- |
| Stanley and Mann (1983) | 11 suicides | | Not reported | Hanging (n = 5)  Gunshot wound (n = 3)  Trauma from falling (n = 2)  Stabbing (n = 1) | 35 (3) | Yes | 9:2 | Yes | Not reported | Matched for post-mortem delay;  FC samples were dissected then stored at –80 °C;  Storage time not reported;  Homogenised tissue;  [3H]spiroperidol (i.e., spiperone) at 0.1–7 nM;  Incubation time and temperature not reported  N.B. Kd of spiperone is 0.5 nM in human cortex (Stanley and Mann, 1983) | 44% ↑ in FC (BA 8 and 9) | (↑) in FC | Not reported | | |
|  | 11 controls | | Not reported | Cardiovascular disease (n = 4)  Road traffic accident (n = 3)  Gunshot wound (n = 3)  Fall (n = 1) | 33 (5) |  | 9:2 |  | Not reported |  |  |  |  |  |  |
| Crow et al. (1984) | 10 suicides | | History of depressive illness (n = 7)    Not reported (n = 3) | “Traumatic” means (n = 4)  BZD OD (n = 3)  AP OD (n = 1)  Analgesic OD (n = 1)  Drowning (n = 1) | 49.8 (20.2) | Yes | 4:6 | Not reported | Not reported | Post-mortem delay not reported;  Brains removed whole and stored at –40 °C;  Storage time not reported;  Homogenised tissue;  [3H]ketanserin at 1 nM;  30 min at room temperature | (↓) in FC | Not reported | | Not reported | |
|  | 19 controls | | Not reported | Died of “various causes in a general hospital” | 56.2 (14.6) |  | 12:7 |  | Not reported |  |  |  |  | – | |
| Mann et al. (1986) | 21 suicides | | No psychiatric information given  No neuropathological evidence of neurological disease or hypoxic damage | Medical examiner determined suicide as cause of death –  Hanging (n = 7)  Gunshot wound (n = 5)  Trauma from falling (n = 4)  OD (n = 2)  Drowning (n = 2)  Stabbing (n = 1) | 36.4 (5.0) | Yes | 17:4 | Yes | Not reported | Matched for post-mortem delay;  Entire frontal cortex was removed and stored at –70 °C;  Similar storage time between groups;  Homogenised tissue;  [3H]spiperone at 0.1–3 nM;  15 min at 37 °C | 28% ↑ in FC | No group difference | Not reported | | |
|  | 21 controls | | No psychiatric information given  No neuropathological evidence of neurological disease or hypoxic damage | Died from non-neurological causes –  Trauma (n = 15)  Ischaemic heart disease (n = 6) | 32.1 (2.0) |  | 18:3 |  | Not reported |  |  |  | – | | |
| Owen et al. (1986) | 19 suicides | | Depression, definite (n = 3)  Depression, possible (n = 6)  Schizophrenia (n = 4)  Personality disorder (n = 2)  No diagnosis (n = 4) | Not reported | 44.7 (4.4) | Yes | 9:10 | Yes | Some but not all patients were taking ADs at the time of death | Matched for post-mortem delay;  Stored at –45 °C;  Not matched for storage time (but no correlation with binding);  ? Homogenised tissue;  [3H]ketanserin at 1 nM;  20 min at 37 °C | (↓) in FC and OC | Not reported | | ND between suicides with and without depression  ND between suicides treated with ADs or not | |
|  | 19 controls | | Not reported | Not reported | 54.2 (4.4) |  | 10:9 |  | Not reported |  |  |  |  | – | |
| Cheetham et al. (1988) | 19 MDD suicides  **Including 13 AD-free MDD suicides** | | Beskow group 1 or 2 depression, determined by psychiatrist using records and interviews  No history of schizophrenia, personality disorder, alcohol or drug abuse or epilepsy | Hanging (n = 5)  CO poisoning (n = 5)  OD (n = 5)  Wounding (n = 2)  Drowning (n = 1) | 40 (3) | Yes | 9:4 | Yes | **In AD-free MDD subgroup: no AD or other psychoactive drugs “recently” – no evidence on blood toxicology**  In remaining 6 patients:  AD only (n = 3)  ADs and BZD (n = 2)  BZD only (n = 1) | ND between groups for post-mortem delay;  Brains stored at –80 °C at autopsy;  ND between groups for storage time;  Homogenised tissue;  [3H]ketanserin at 0.07–5.4 nM;  15 min at 37 °C; | **23% ↓ in HC in AD-free subgroup vs. controls**  (↓) in TC, OC and HC in all patients vs. controls and in AD-free subgroup vs. controls  (↑) in FC in all patients vs. controls and in AD-free subgroup vs. controls | ↑ in FC, TC and OC in all patients vs. controls  ↑ in FC in AD-treated subgroup vs. controls  (↑) in FC, TC, OC and HC in AD-free subgroup vs. controls | | **No effect of medication status on binding values**  ND between suicides who died by violent vs. non-violent means | |
|  | 13 controls | | Sudden death from non-CNS causes  No documented evidence of mental illness | Myocardial infarction (n = 16)  Fall (n = 1)  Accidental CO poisoning (n = 1)  Drowning (n = 1) | 39 (3) |  | 9:4 |  | Not reported |  |  |  |  | – | |
| Arora et al. (1989) | 32 suicides | | Police and coroner’s records insufficient to assign psychiatric diagnoses | Hanging (n = 12)  Shooting (n = 7)  CO poisoning (n = 6)  OD (n = 5)  Stabbing (n = 1)  Jumping from height (n = 1)  As determined by the medical examiner | 48.1 (20.5) | Yes | 22:10 | Yes | Only 1 patient had evidence of recent AD use (amitriptyline/nortriptyline) on blood toxicology | Matched for post-mortem delay;  Storage details not reported;  Storge time not reported;  Homogenised tissue;  [3H]spiperone;  20 min at 37 °C | ↑ in FC | (↑) in FC | | Not reported | |
|  | 37 controls | | Not reported | Myocardial infarction or other heart disease (n = 18)  Pulmonary embolism or other pulmonary disease (n = 7)  CO poisoning (n = 4)  Road traffic and other accidents (n = 4)  Renal failure (n = 2)  Asthma (n = 1)  Asphyxia (n = 1)  As determined by the medical examiner | 45.3 (16.9) |  | 25:12 |  | Not reported |  |  |  |  | – | |
| Arango et al. (1990) | 11 suicides | | No psychiatric information given  No evidence of chronic alcoholism | Violent means –  Fall from height (n = 4)  By firearm (n = 2)  Hanging (n = 2)  CO poisoning (n = 1)  Drowning (n = 1)  By subway (n =1) | 43.55 (20.45) calculated | Yes | 9:2 | Yes | Not reported | Matched for post-mortem delay;  Cerebral hemisphere was cut into 1 cm coronal slices then stored at –70 °C;  Storage time not reported;  Sectioned slides and homogenised tissue;  [125I]LSD at 2 nM;  15–120 min, temperature unclear  N.B. K_d_ of LSD is 1.26 nM in human cortex (Diez-Alarcia et al., 2021) | ↑ in PFC  (↑) in TC | (↑) in PFC  (↑) in TC | | Not reporte | |
|  | 11 controls | | No psychiatric information given  No chronic alcoholism, CNS disease or AIDS | Cardiac arrest (n = 4)  By firearm, homicide (n = 2)  By firearm, accidental (n = 1)  Laceration, homicide (n = 1)  Renal failure (n = 1)  Liver failure (n = 1)  Respiratory failure (n = 1) | 42.82 (19.38) calculated |  | 9:2 |  | Not reported |  |  |  |  | Binding values were not associated with violent deaths in the control group | |
| Gross-Isseroff et al. (1990) | 12 suicides | | No schizophrenia, alcoholism or drug abuse  No neuropathology | Hanging (n = 3)  Jumping from height (n = 3)  Suffocation (n = 2)  Gunshot (n = 1)  Unknown (but considered to be suicide) (n = 3) | 45.75 (18.64) | Yes | 7:5 | Yes | Not reported  Toxicology completed for some psychotropic drugs; brain tissue assayed for presence of TCAs | Matched for post-mortem delay;  Storage time not reported;  2.5 cm blocks were dissected and stored at –70 °C;  Sectioned slides;  [3H]ketanserin at 1.5 nM;  120 min at 25 °C | ↓ in MFG and SFG in young suicides vs. young controls  ↓ in HC in old suicides vs. old controls  (↓) in most prefrontal areas in all patients vs. controls | ND | | Not reported | |
|  | 12 controls | | No schizophrenia, alcoholism or drug abuse  No neuropathology | Multiple trauma (n = 6)  Heart failure (n = 2)  Burns (n = 1)  Gastrointestinal bleeding (n = 1)  Drowning (n = 1)  Food poisoning (n = 1) | 45.42 (21.17) |  | 7:5 |  | Not reported  Toxicology completed for some psychotropic drugs; brain tissue assayed for presence of TCAs |  |  |  |  | – | |
| Arango et al. (1992) | 13 suicides | | Not reported | Not reported | 38.9 (3.9) combined | Yes | Not reported | Yes | Toxicological tests (blood, bile, urine ± brain) were negative for psychotropics | Matched for post-mortem delay;  Brain was sectioned into 15 µm slices then stored at –70 °C;  Storage time not reported;  Sectioned slides;  [125I]LSD at 2 nmol/L;  90 min at 21 °C | 49–67% ↑ in PFC    (↑) in TC | Not reported | | Not reported | |
|  | 13 controls | | Not reported | Not reported |  |  | Not reported |  | Toxicological tests (blood, bile, urine ± brain) were negative for psychotropics |  |  |  |  | - | |
| Hrdina et al. (1993) | 10 depressed suicides | | DSM-III for unipolar MDE (n = 7) or BPAD depression (n = 3), by case note review | Hanging (n = 8)  Jumping from height (n = 1)  CO poisoning (n = 1) | 50.8 (13.4) | Yes | 8:2 | Yes | **No ADs for at least 2 wk**  **No psychotropics thought to interact with 5-HT_2A_**  No toxicological analyses were completed | Matched for post-mortem delay;  Blocks of 8–10 by 8–10 mm were cut from PFC and then stored at –70 °C;  Storage time not reported;  Homogenised tissue;  [3H]ketanserin;  15 min at 37 °C | 67% ↑ in PFC | (↑) in PFC | | Not reported | |
|  | 10 controls | | No documented evidence of mental illness  No history of schizophrenia, alcohol or drug abuse or epilepsy | Acute myocardial infarction (n = 7)  Acute heart failure (n = 1)  Fatal accident (n = 1) | 49.7 (4.4) |  | 8:2 |  | No psychotropics thought to interact with 5-HT_2A_  No toxicological analyses were completed |  |  |  |  | – | |
| Arranz et al. (1994) | 18 suicides | | 7 patients had a clear history of depressive symptoms prior to death  No history of alcohol abuse | Medical examiner stated suicide as cause of death –  Violently (hanging or gunshot wound) (n = 8)  CO poisoning (n = 5)  TCA and BZD OD (n = 3)  AP and BZD OD (n = 1)  Analgesics OD (n = 1) | 43.0 (4.8) | Yes | 12:6 | Yes | Evidence of psychoactive medication use | Matched for post-mortem delay;  Frontal areas were dissected and stored at –70 °C;  Storage time not reported;  Homogenised tissue;  [3H]ketanserin at 0.2–4 nmol/L;  180 min at 20 °C | (↑) in FC (BA 9, 10 and 11) | (↑) in FC | | Not reported | |
|  | 23 controls | | No documented history of mental or CNS disorder | Ischaemic heart disease Pulmonary disease  Accidental drowning  Road traffic accident  Accidental CO poisoning | 49.4 (3.8) |  | 19:4 |  | No psychoactive medication use |  |  |  |  | – | |
| Lowther et al. (1994) | 73 suicides | | **20 patients were AD-free MDD**  28 patients were AD-treated MDD  25 patients had another or no psychiatric diagnosis | Hanging (n = 26)  OD (n = 24)  CO poisoning (n = 11)  Stab wound (n = 2)  Jumping from height (n = 6)  Impact with a moving vehicle (n = 2)  Burns (n = 1)  Suffocation (n = 1) | 48 (2) | Yes | 54:19 | Yes | **No ADs for at least 3 mo** | Matched for post-mortem delay;  Brains were stored at –80 °C;  Matched for storage time;  Homogenised tissue;  [3H]ketanserin at 0.03–4 nM;  15 min­ at 37 °C | (↓) in FC, TC, and HC  No group difference in PC  **(↓) in FC, TC and HC in AD-free suicides vs. controls**  **(↑) in PC in AD-free suicides vs. controls** | (↑) in FC and PC  ↑ in TC and HC  **(↑) in FC and PC in AD-free suicides vs. controls**  **(↓) in HC in AD-free suicides vs. controls**  **No group difference in TC in AD-free suicides vs. controls** | | ND in binding values between patients who died of violent vs. non-violent means;  **ND in binding between AD-free patients who died of violent vs. non-violent means** | |
|  | 70 controls | | No documented evidence of mental illness | Myocardial infarction (n = 55)  Road traffic accident (n = 4)  Acute asthma (n = 2)  Accidental drowning (n = 2)  Ruptured aneurysm (n = 1)  CO poisoning (n = 1)  Fall (n = 1)  Pulmonary embolism (n = 1)  Pulmonary thrombosis (n = 1) Arterial thrombosis (n = 1)  Electrocution (n = 1) | 47 (2) |  | 53:17 |  | Not reported |  |  |  |  | – | |
| Stockmeier et al. (1997) | 10 depressed suicides  (from a larger suicide cohort) | | DSM-III-R for MDE, by case note review  No current substance use disorder  Within larger suicide cohort:  Dysthymia (n = 2)  Parkinson’s disease (n = 1) | Within larger suicide cohort:  Hanging (n = 4)  Gunshot to chest (n = 3)  Gunshot to head (n = 2)  Stab/slash (n = 2)  OD (n = 1)  CO poisoning (n = 1) | 55 (19) | Yes | Within larger suicide cohort:  10:3 | Yes | Within larger suicide cohort:  ADs (n = 6 max.)  BZD (n = 3 max.)  No patients were on APs  According to medication histories and blood and urine toxicology | Matched for post-mortem delay;  Tissue blocks were dissected then stored at –80 °C;  Storage time was significantly longer in patients;  Sectioned slides;  [3H]ketanserin at 2 nM;  60 min at room temperature | (↑) in Rt PFC (BA 10) and Rt HC | Not reported | | None found – post-hoc exclusion of 3 patients with recent AD prescriptions did not alter results  Otherwise not reported | |
|  | 9 controls for PFC analyses; 10 for HC analyses  (from a larger control cohort) | | No evidence of psychiatric illness, as per family interview and/or coroner’s records  No current substance use disorder | Within larger control cohort:  Heart disease (n = 10)  Aneurysm (n = 2)  Road traffic accident (n = 1)  Gunshot (n = 1)  Smoke inhalation (n = 1) | 57 (18) |  | Within larger control cohort:  12:3 |  | Within larger control cohort:  No controls were taking ADs, APs, BZDs or other psychotropic medication  According to medication histories and blood and urine toxicology |  |  |  |  | – | |
| *Rosel et al. (1998)* | ***17 AD-free MDD violent suicides*** | | ***DSM-III-R depressive symptoms, from joint psychiatrist–coroner interview with relative***  ***No schizophrenia, personality disorder, alcohol/drug abuse or epilepsy*** | ***Forensic pathologist stated suicide as cause of death –***  ***Hanging (n = 9)***  ***Gunshot (n = 4)***  ***Knife wound (n = 4)*** | ***42 (4.8)*** | ***Yes*** | ***13:4*** | ***Yes*** | ***“None … had taken any antidepressant drugs prior to death”*** | ***Matched for post-mortem delay;***  ***Cortical regions were dissected then stored at –80 °C;***  ***Storage time not reported;***  ***Homogenised tissue;***  ***[3H]ketanserin at 0.3–10 nM;***  ***180 min at 25 °C*** | ***↓ in HC***  ***(↓) in FC***  ***(↑) in CC*** | ***↓ in HC***  ***(↓) in FC***  ***(↑) in CC*** | | ***5-HT_2A_ binding was negatively correlated with 5-HTT binding in the control but not patient group***  ***No correlation found between 5-HT_2A_ binding and suicide method*** | |
|  | *17 controls* | | *No psychiatric or neurological disease, corroborated by joint psychiatrist–coroner interview with relative* | *Ischaemic heart disease (n = 12)*  *Road traffic accident (n = 5)* | *49 (4.0)* |  | *15:2* |  | *Not reported* |  |  |  |  | *­–* | |
| Turecki et al. (1999) | 11 suicides  (from a large genetic study) | | No psychiatric information given | Within larger suicide cohort:  Hanging (n = 26)  Other methods (n = 11)  CO poisoning (n = 8)  Shooting (n = 6)  OD (n = 5) | 36.09 (10.75) | Yes | Not reported | Yes | Not reported | Matched for post-mortem delay;  Dissected “according to standard procedures” then stored at –80 °C;  Storage time not reported;  Homogenised tissue;  [3H]ketanserin;  Incubation time and temperature not reported | ↑ in PFC (BA 8 and 9) | ND between groups (values not given) | | Not reported | |
|  | 11 controls  (from a large genetic study) | | Not reported | Cardiovascular disease (n = 7)  Work related accident or non-impulsive road traffic accident (n = 4) | 33.54 (9.95) |  | Not reported |  | Not reported |  |  |  |  | – | |
| *Rosel et al. (2000)* | ***18 AD-free MDD violent suicides*** | | ***DSM-III-R depressive symptoms, from joint psychiatrist–coroner interview with relative***  ***No schizophrenia, personality disorder, alcohol/drug abuse or epilepsy*** | ***Forensic pathologist stated suicide as cause of death –***  ***Hanging (n = 9)***  ***Gunshot (n = 4)***  ***Knife wound (n = 4)*** | ***42 (4.8)*** | ***Yes*** | ***14:4*** | ***Yes*** | ***No ADs for at least 6 mo***  ***Never taken lithium or APs*** | ***Matched for post-mortem delay;***  ***Cortical regions were dissected then stored at –80 °C;***  ***Storage time not reported;***  ***Homogenised tissue;***  ***[3H]ketanserin at 0.3–10 nM;***  ***180 min at 25 °C*** | ***↓ in HC, associated with ↑[IP_3_]***  ***(↓) in FC, associated with (↓)[IP_3_]*** | ***↓ in HC***  ***(↓) in FC*** | | ***Not reported*** | |
|  | *18 controls* | | *No psychiatric or neurological disease, corroborated by joint psychiatrist–coroner interview with relative* | *Ischaemic heart disease (n = 12)*  *Road traffic accident (n = 6)* | *49 (4.0)* |  | *15:3* |  | *Not reported* |  |  |  |  | *–* | |
| Pandey et al. (2002) | 15 teenage suicides | | Various DSM-III-R diagnoses  Brains had no neuropathological abnormalities or HIV antibodies | Medical examiner determined suicide as cause of death –  Hanging (n = 7)  Gunshot wound (n = 4)  OD (n = 3)  Asphyxia (n = 1) | 15.13 (1.96) | Yes | 9:6 | Yes | ADs (n = 2)  Methylphenidate (n = 1)  Other patients had other positive toxicology results | Matched for post-mortem delay;  Storage details or time not reported;  Homogenised tissue;  [125I]LSD at 0.25–3 nM;  90 mins at 37 °C | ↑ in Rt PFC | (↑) in Rt PFC | | Not reported | |
|  | 15 controls | | “psychiatrically normal”  Brains had no neuropathological abnormalities or HIV antibodies | Gunshot wound (n = 8)  Accident (multiple injuries) (n = 2)  Drowning (n = 2)  Stab wounds (n = 1)  Hanging (n.= 1)  Cardiac arrythmia (n = 1) | 17.00 (1.77) |  | 12:3 |  | No psychotropic drugs found on toxicology |  |  |  |  | – | |
| Rosel et al. (2004) | **19 AD-free MDD suicides** | | **DSM-IV for MDD, from joint psychiatrist–coroner interview with relative**  **No history of schizophrenia, personality disorder, alcohol or drug abuse or epilepsy** | **Forensic pathologist stated suicide as cause of death –**  **Hanging (n = 10)**  **Gunshot (n = 5)**  **Knife wound (n = 4)** | **42 (4.8)** | **Yes** | **15:4** | **Yes** | **No AD or AP for at least 6 mo**  **No relevant non-psychotropic medication** | **Matched for post-mortem delay;**  **Cortical regions were dissected then stored at –80 °C;**  **Storage time was up to 6 mo;**  **Homogenised tissue;**  **[3H]ketanserin at 0.3–10 nM;**  **180 min at 25 °C** | **↓ in HC, associated with ↑[IP_3_]**  **(↓) in FC, associated with (↓)[IP_3_]** | **↓ in HC** | | **Not reported** | |
|  | 19 controls | | No psychiatric or neurological disease, corroborated by joint psychiatrist–coroner interview with relative | Ischaemic heart disease (n = 12)  Road traffic accidents (n = 7) | 49 (3.7) |  | 15:4 |  | No relevant non-psychotropic medication |  |  |  |  | – | |
| Underwood et al. (2012) | **15 AD-free suicides** | | **Major depression (n = 12)**  **Personality disorder (n = 2)**  **Schizoaffective disorder (n = 1)**  **Determined by psychological autopsy** | **Hanging (n = 8)**  **Gunshot wound (n = 4)**  **Fall from height (n = 3)** | **45 (5)** | **Yes** | **10:5** | **Yes** | **Toxicological tests (blood, urine, bile and vitreous humor) were negative for ADs and APs** | **Binding did not correlate with post-mortem delay;**  **Rt hemicerebrum was cut into 2cm coronal sections then stored at –80 °C;**  **Storage time not reported;**  **Sectioned slides;**  **[3H]ketanserin at 2 nM;**  **Incubation time and temperature not reported** | **(↓) in dorsal PFC (BA 9 sulcus and gyrus) and ventral PFC (BA 47 gyrus)**  **(↑) in ventral PFC (BA 47 sulcus)** | **Not reported** | | **Not reported** | |
|  | 15 controls | | “Normal, non-psychiatric controls”, determined by psychological autopsy | Cardiovascular disease (n = 10)  Road traffic accident (n = 5) | 46 (5) |  | 10:5 |  | Toxicological tests (blood, urine, bile and vitreous humor) were negative for ADs and APs |  |  |  |  | – | |
| *Muguruza et al. (2013)* | *13 suicides* | | *Reactive depression (n = 3)*  *Anxiety disorder (n = 2)*  *Dysthymia (n = 2)*  *Neurotic depression (n = 1)*  *Anorexia nervosa (n = 1)*  *Personality disorder (n = 1)*  *Reactive depression and personality disorder (n = 1)*  *Personality disorder and sedative dependence (n = 1)*  *Alcohol dependence (n = 1)* | *Not reported* | *46 (3)* | *Yes* | *9:4* | *Yes* | *BZD only (n = 4)*  *Venlafaxine and metamizole (n = 1)*  *Fluoxetine and BZD (n = 1)*  *Trazodone and furosemide (n = 1)*  *BZD and diphenhydramine (n = 1)* | *Matched for post-mortem delay;*  *0.5–1 g sections of PFC were dissected then stored at –80 °C;*  *Mean storage time was 110 mo for patients and 93 mo for controls;*  *Homogenised tissue;*  *[3H]ketanserin at 10 nM;*  *60 min at 37 °C* | *(↑) in PFC (BA 9)* | *Not reported* | | *Not reported* | |
|  | 13 controls | | No neuropsychiatric disorder  No drug abuse | Sudden and unexpected deaths | 44 (3) |  | 9:4 |  | Toxicological tests (blood, urine, liver and gastric contents) were negative for psychotropic drugs |  |  |  | |  | |

↑ and ↓ denote significantly higher or lower values in completed suicides relative to controls, respectively. (↑) and (↓) denote non-significantly higher or lower values in completed suicides relative to controls, respectively. °C, degrees Celsius; ACC, anterior cingulate cortex; AD(s), antidepressant(s); AIDS, acquired immune deficiency syndrome; AP(s), antipsychotics; BA, Brodmann area; BPAD, bipolar affective disorder; CNS, central nervous system; CO, carbon monoxide; dlPFC, dorsolateral prefrontal cortex; DSM-III(-R), Diagnostic and Statistical Manual version 3 (Revised); FC, frontal cortex; g, gram(s); HC, hippocampus; [IP_3_], concentration of inositol triphosphate; K_d_, equilibrium dissociation constant; LSD, lysergic acid diethylamide; MDD, major depressive disorder; MDE, major depressive episode; mo, month(s); ND, no statistically significant difference; nM, nanomole; OC, occipital cortex; OD, deliberate drug overdose; PC, parietal cortex; PFC, prefrontal cortex; Rt, right; TC, temporal cortex; TCA(s), tricyclic antidepressant(s). **Details in bold font describe findings in MDD patients who were antidepressant-free at death**. *Studies in italicised font were excluded from meta-analyses (subject groups in Rosel et al. 2004 mostly comprise subjects from Rosel et al. 1998 and 2000; complete data were unavailable for Muguruza et al. 2013).*

| Type of case–control study | Study | Selection | | | | | | | Comparability | | Exposure | | | | Agency for Healthcare Research and Quality (AHRQ) rating |
| --- | --- | --- | --- | --- | --- | --- | --- | --- | --- | --- | --- | --- | --- | --- | --- |
|  |  | Case definition | Representat-iveness of cases | | Selection of controls | | | Definition of controls | Age matching/ adjustment | Gender matching/ adjustment | Ascertainment of exposure; same method used for all subjects? | | Non-response rate | |  |
|  | | | | | | | | | | | | | | | |
| *PET/SPECT studies* | D’haenen et al. (1992) | ☆ | | – | | – | – | | ☆ | – | – | – | | ☆ | Poor |
|  | Attar-Lévy et al. (1999) | ☆ | | ☆ | | – | ☆ | | ☆ | – | ☆ | – | | ☆ | Good |
|  | Meyer et al. (1999) | ☆ | | – | | ☆ | ☆ | | ☆ | – | ☆ | ☆ | | ☆ | Good |
|  | Yatham et al. (2000) | ☆ | | – | | – | ☆ | | ☆ | – | ☆ | – | | ☆ | Fair |
|  | Meyer et al. (2001) | ☆ | | – | | ☆ | ☆ | | ☆ | – | ☆ | ☆ | | ☆ | Good |
|  | Messa et al. (2003) | ☆ | | – | | – | ☆ | | ☆ | – | ☆ | ☆ | | ☆ | Fair |
|  | Mintun et al. (2004) | ☆ | | – | | – | ☆ | | ☆ | – | ☆ | – | | ☆ | Fair |
|  | Sheline et al. (2004) | ☆ | | – | | ☆ | ☆ | | ☆ | – | ☆ | – | | ☆ | Good |
|  | Baeken et al. (2011) | ☆ | | – | | – | ☆ | | ☆ | ☆ | ☆ | ☆ | | ☆ | Fair |
|  | Baeken et al. (2012) | ☆ | | – | | – | ☆ | | ☆ | ☆ | – | – | | ☆ | Poor |
|  | Erritzoe et al. (2022) | ☆ | | – | | – | ☆ | | ☆ | – | ☆ | ☆ | | ☆ | Fair |
|  | | | | | | | | | | | | | | | |
| *Post-mortem studies* | Stanley & Mann (1983) | ☆ | | – | | ☆ | ☆ | | ☆ | ☆ | ☆ | ☆ | | ☆ | Good |
|  | Crow et al. (1984) | – / ☆ | | – / – | | – / – | – / – | | – / – | – / – | ☆ / ☆ | – / – | | ☆ / ☆ | Poor / poor |
|  | Mann et al. (1986) | ☆ | | – | | – | ☆ | | ☆ | ☆ | ☆ | ☆ | | ☆ | Fair |
|  | Owen et al. (1986) | – | | – | | – | – | | – | – | – | – | | ☆ | Poor |
|  | Cheetham et al. (1988) | ☆ / ☆ | | – / – | | – / – | ☆ / ☆ | | ☆ / ☆ | ☆ / ☆ | ☆ / ☆ | – / – | | ☆ / ☆ | Fair / fair |
|  | Arora et al. (1989) | ☆ | | – | | ☆ | ☆ | | ☆ | ☆ | ☆ | ☆ | | ☆ | Good |
|  | Arango et al. (1990) | ☆ | | – | | – | ☆ | | ☆ | – | ☆ | – | | ☆ | Fair |
|  | Gross-Isseroff et al. (1990) | ☆ | | – | | ☆ | ☆ | | ☆ | ☆ | – | – | | ☆ | Poor |
|  | Arango et al. (1992) | – | | – | | – | – | | ☆ | ☆ | – | – | | ☆ | Poor |
|  | Hrdina et al. (1993) | ☆ | | – | | ☆ | ☆ | | ☆ | ☆ | – | – | | ☆ | Poor |
|  | Arranz et al. (1994) | – / ☆ | | – / – | | ☆ / ☆ | ☆ / ☆ | | ☆ | – / – | – / ☆ | – / – | | ☆ / ☆ | Poor / good |
|  | Lowther et al. (1994) | ☆ / ☆ | | – / – | | – / – | – / – | | ☆ / ☆ | ☆ / ☆ | ☆ / ☆ | – / ☆ | | ☆ / ☆ | Poor / poor |
|  | Stockmeier et al. (1997) | ☆ | | – | | ☆ | ☆ | | ☆ | – | – | – | | ☆ | Poor |
|  | Turecki et al. (1999) | ☆ | | – | | ☆ | ☆ | | ☆ | ☆ | – | – | | ☆ | Poor |
|  | Pandey et al. (2002) | ☆ | | – | | – | – | | – | – | ☆ | – | | ☆ | Poor |
|  | Rosel et al. (2004) | ☆ / ☆ | | – / – | | – / – | ☆ / ☆ | | ☆ / ☆ | ☆ / ☆ | ☆ / ☆ | – / – | | ☆ / ☆ | Fair / fair |
|  | Underwood et al. (2012) | ☆ | | – | | ☆ | ☆ | | ☆ | ☆ | – | – | | ☆ | Poor |
|  | Dean et al. (2014) | ☆ | | – | | – | ☆ | | ☆ | ☆ | ☆ | ☆ | | ☆ | Fair |
|  | Muguruza et al. (2014) | ☆ | | – | | – | ☆ | | ☆ | ☆ | ☆ | ☆ | | ☆ | Fair |

*Supplementary Table 3*. **Results of Newcastle–Ottawa risk of bias assessments of included case–control studies**. Studies were scored on selection (maximum 4 stars), comparability (maximum 2 stars) and exposure (maximum 2 stars). These scores were then converted to a corresponding Agency for Healthcare Research and Quality (AHRQ) rating of “good”, “fair” or “poor”, based on previously published thresholds (Shamsrizi et al. 2020, *BMJ Open*). Studies in depressed suicide victims are given two scores for each category – for quality with respect to depression and suicide, respectively.

*Supplementary Table 4* – in MDD patients, does antidepressant treatment lead to a change in 5-HT_2A_ binding and is this associated with clinical outcome?

| Study | Sample (at baseline) | Diagnosis | Mean HDRS (SD) | Mean age (SD) | Gender (M:F) | Medication / antidepressant status | Imaging details and radioligand | Outcome summary: change in binding following treatment (± clinical response) |
| --- | --- | --- | --- | --- | --- | --- | --- | --- |
| Attar-Lévy et al. (1999) | 7 drug-free MDD | DSM-III for MDE without psychotic symptoms | 54 (6) at baseline | 40 (11) | 3:4 | No ADs for at least 1 yr, except 1 patient who stopped fluoxetine 2 wk prior to study  Then given clomipramine (150 mg/day) for at least 3 wk | PET – [18F]setoperone  Baseline scan: prior to first clomipramine dose  Follow-up scan: after 21-39 d (mean 26 d, SD 6 d) of clomipramine – time between last dose and scan not reported | ↓ in FC, TC, PC and OC  Mean 18% reduction in cortical binding  Significant 70% reduction in mean HDRS scores |
| Yatham et al. (1999) | 10 AD-free MDD | DSM-III-R for MDE (and DSM-IV for MDD) | 27.0 (5.6) at baseline  7.0 (7.8) following desipramine | 40.6 (9.2) | 5:5 | No psychotropics except BZDs for at least 2 wk  Never received ECT  Then given desipramine (mean dose 160 mg) for 3-4 wk | PET – [18F]setoperone  Baseline scan: prior to first desipramine dose  Follow-up scan: after 3-4 wk of desipramine – 12-20 hr after last dose | ↓ in FC, TC, PC and OC  8.1% reduction in binding across entire cluster  Response (minimum 50% reduction in HDRS score) in 8 patients  Change in HDRS score (or its suicide subscale score) did not correlate with change in binding |
| Moresco et al. (2000) | **9 antidepressant-naïve MDD** | **DSM-IV for MDE**  **No other axis I diagnosis**  **No birth or head trauma or other neurological disease** | **27 (7) at baseline**  **9 (9) after 6 wk of fluvoxamine** | **39.2 (9.128) calculated** | **4:5** | **Never ADs, APs, lithium, AEDs, other mood stabilisers or chronic BZDs (some patients were taking an acute course of BZDs)**  **Then given fluvoxamine (uptitrated to 150mg twice daily) for at least 6 wk** | **PET – [18F]FESP**  **Baseline scan: prior to first fluvoxamine dose (same day)**  **Follow-up scan: after 4-6 wk of fluvoxamine – time between last dose and scan not reported** | **↑ in FC and OC**  **Mean 31% increase in cortical binding**  **Response (minimum 50% reduction in HDRS score) within 6 wk for 8 patients**  **Greater ↑ in early HDRS responders than late HDRS responders** |
| Meyer et al. (2001) | 19 drug-free unipolar MDD | DSM-IV for MDE secondary to MDD  No psychotic or bipolar symptoms and no other axis I diagnosis  No history of alcohol or drug abuse | 21.8 (3.8) | 30.8 (6.1) | 12:7 | No psychotropics for at least 3 mo  No ADs for at least 6 mo  Then given paroxetine (20 mg/day) for at least 6 wk | PET – [18F]setoperone  Baseline scan: prior to first paroxetine dose  Follow-up scan: after 6 wk of paroxetine –11-14 hr after last dose | Age-dependent ↓ in MFC, lOFC, PHG, posteromedial TC and rostral ACC:  10% ↓ in 20-30 yo  0% difference in 30-40 yo  10 responders, 6 partial responders, 3 non-responders  No difference in binding between responders and remaining subjects (partial and non-responders) |
| *Yatham et al. (2010)* | *15 AD-free treatment resistant MDD* | *DSM-IV for MDD*  *No other axis I diagnosis, substance or alcohol misuse within last 6 mo* | *33.6 (7.71) at baseline*  *11.26 (10.2) following course of ECT* | *44.26 (10.4)* | *6:9* | *No psychotropics except BZDs for at least 1 wk (and at least 5 half-lives)*  *Then given a course of ECT* | *PET – [18F]setoperone*  *Baseline scan: before first ECT session*  *Follow-up scan: within 1 wk of last ECT session* | *↓ in Rt MFG, Rt lingual gyrus, Rt PHG and Lt lateral occipital gyrus following ECT*  *3.8% reduction in binding across entire cluster*  *Significant reduction in mean HDRS score following course of ECT (p < 0.0001)*  *Response (minimum 50% reduction in HDRS) in 10 patients*  *A significant correlation (p = 0.001) between change HDRS score and change in binding in Rt mOFC, Rt PHG and Rt lingual gyrus did not survive correction for multiple comparisons (p = 0.08–0.10)* |
| *Baeken et al. (2011)* | *14 AD-free treatment resistant MDD* | *MINI for MDE*  *No alcohol or drug dependence*  *No suicide attempts during current episode of illness*  *No history of epilepsy, neurosurgery or having metal or magnetic objects in the brain* | *25.57 (3.92) at baseline* | *45.3 (11.7)* | *8:13* | *Free of all psychotropics (except BZDs) for at least 2 wk*  *Then given 10 HF-rTMS sessions (to Lt dlPFC)* | *SPECT – [123I]5-I-R91150*  *Baseline scan: within 2 d of starting HF-rTMS treatment*  *Follow-up scan: within 1 wk of last HF-rTMS session* | *↓ in Rt and Lt dlPFC*  *↑ in Lt HC*  *(↑) in Rt HC*  *Response (minimum 50% reduction in HDRS) in 9 patients (of original 21 patients)*  *Significant positive correlation between change in HDRS score and change in binding in Rt and Lt dlPFC*  *Significant negative correlation between change in HDRS score and change in binding in Rt HC* |

↑ and ↓ denote significantly higher or lower 5-HT_2A_ binding after relative to before antidepressant treatment, respectively. (↑) and (↓) denote non-significantly higher or lower 5-HT_2A_ binding after relative to before antidepressant treatment, respectively. ACC, anterior cingulate cortex; AD(s), antidepressant(s); AP(s), antipsychotics; BZDs, benzodiazepines; d, day(s); dlPFC, dorsolateral prefrontal cortex; DSM III(-R), Diagnostic and Statistical Manual version 3 (revised); DSM-IV, Diagnostic and Statistical Manual version 4; ECT, electroconvulsive therapy; FC, frontal cortex; HC, hippocampus; HDRS, Hamilton Depression Rating Scale; HF-rTMS, high frequency repetitive transcranial magnetic stimulation; hr, hour(s); lOFC, lateral orbitofrontal cortex; Lt, left; MDD, major depressive disorder; MDE, major depressive episode; MFC, medial frontal cortex; MFG, medial frontal gyrus; MINI, Mini-International Neuropsychiatric Interview; mo, month(s); mOFC, medial orbitofrontal cortex; mPFC, medial prefrontal cortex; OC, occipital cortex; PC, parietal cortex; PET, positron emission tomography; PHG, parahippocampal gyrus; Rt, right; SPECT, single photon emission computed tomography; TC, temporal cortex; wk, week(s); yr, year(s); yo, years old. **Studies in bold font report full data for MDD cohort who were previously antidepressant-naïve.** *Studies in italicised font looked at the effects of non-pharmacological antidepressant treatments.*

*Supplementary Table 5* – in MDD patients, is 5-HT_2A_ binding prior to antidepressant treatment associated with clinical outcome post-treatment?

| Study | Sample (at baseline) | Diagnosis | Mean HDRS (SD) | Mean age (SD) | Gender (M:F) | Medication status | Imaging details and radioligand | Outcome summary |
| --- | --- | --- | --- | --- | --- | --- | --- | --- |
| Attar-Lévy et al. (1999) | 7 unmedicated MDD | DSM-III for MDE without psychotic symptoms | 54 (6) at baseline | 40 (11) | 3:4 | No ADs for at least 1 yr, except 1 patient who stopped fluoxetine 2 wk prior to study  Then given clomipramine (150 mg/day) for at least 3 wk | PET – [18F]setoperone  Baseline scan: prior to first clomipramine dose  Follow-up scan: after 21-39 d (mean 26 d, SD 6 d) of clomipramine – time between last dose and scan not reported | No correlation between regional binding prior to treatment and subsequent clinical response |
| Meyer et al. (2001) | 19 drug-free unipolar MDD | DSM-IV for MDE secondary to MDD  No psychotic or bipolar symptoms and no other axis I diagnosis  No history of alcohol or drug abuse | 21.8 (3.8) | 30.8 (6.1) | 12:7 | No psychotropics for at least 3 mo  No ADs for at least 6 mo  Then given paroxetine (20 mg/day) for at least 6 wk | PET – [18F]setoperone  Baseline scan: prior to first paroxetine dose  Follow-up scan: after 6 wk of paroxetine –11-14 hr after last dose | No difference in pre-paroxetine binding between responders and remaining subjects (partial and non-responders) |
| *Baeken et al. (2011)* | *14 AD-free treatment resistant MDD* | *MINI for MDE*  *No alcohol or drug dependence*  *No suicide attempts during current episode of illness*  *No history of epilepsy, neurosurgery or having metal or magnetic objects in the brain* | *25.57 (3.92) at baseline* | *45.3 (11.7)* | *8:13* | *Free of all psychotropics (except BZDs) for at least 2 wk*  *Then given 10 HF-rTMS sessions (to Lt dlPFC)* | *SPECT – [123I]5-I-R91150*  *Baseline scan: within 2 d of starting HF-rTMS treatment*  *Follow-up scan: within 1 wk of last HF-rTMS session* | *No correlation between regional binding prior to treatment and clinical response* |

AD(s), antidepressant(s); BP, binding potential; d, day(s); dlPFC, dorsolateral prefrontal cortex; DSM-III, Diagnostic and Statistical Manual version 3; DSM-IV, Diagnostic and Statistical Manual version 4; HDRS, Hamilton Depression Rating Scale; HF-rTMS, high frequency repetitive transcranial magnetic stimulation; hr, hour(s); Lt, left; MDD, major depressive disorder; MDE, major depressive episode; MINI, Mini International Neuropsychiatric Interview; mo, month(s); PET, positron emission tomography; SD, standard deviation; SPECT, single photon emission computed tomography; wk, week; yr, year(s). *Studies in italicised font looked at the effects of non-pharmacological antidepressant treatments.*
